# The Steroidal Profile Modulates Adaptive Immune Response and Prognosis in Adrenocortical Carcinoma: Analysis of TCR and BCR Repertoires

**DOI:** 10.1002/cam4.71781

**Published:** 2026-06-05

**Authors:** Jean Silva de Souza Resende, Igor Samesima Giner, João Carlos Degraf Muzzi, José Alexandre Marzagão Barbuto, Enzo Lalli, Mauro Antônio Alves Castro, Bonald Cavalcante de Figueiredo

**Affiliations:** ^1^ Instituto de Pesquisa Pelé Pequeno Príncipe, Faculdades Pequeno Príncipe Complexo Pequeno Príncipe Curitiba PR Brazil; ^2^ Bioinformatics and Systems Biology Laboratory Federal University of Paraná Curitiba PR Brazil; ^3^ Department of Immunology, Institute of Biomedical Sciences University of Sao Paulo São Paulo SP Brazil; ^4^ Laboratory of Medical Investigation in Pathogenesis and Targeted Therapy in Onco‐Immuno‐Hematology (LIM‐31), Department of Hematology, Hospital das Clínicas HCFMUSP, Faculty of Medicine, University of São Paulo São Paulo SP Brazil; ^5^ Institut de Pharmacologie Moléculaire et Cellulaire CNRS UMR7275, Inserm U1323, Université Côte D'azur Valbonne France

**Keywords:** immunoinformatics, immunosuppression, prognosis, steroidal phenotype, tumor microenvironment

## Abstract

Adrenocortical carcinoma (ACC) is a rare and aggressive malignant neoplasm with limited therapeutic options and an often poor prognosis. A deeper understanding of its interaction with the immune system is essential for the advancement of personalized treatment strategies, particularly in light of the tumor heterogeneity of hormone production. ACC tumors can be classified into high steroid phenotype (HSP) and low steroid phenotype (LSP) subtypes, which exhibit distinct biological behaviors and immunological microenvironments. However, the composition and prognostic significance of T‐cell and B‐cell receptor (TCR and BCR) repertoires in these subtypes remain largely unknown. In this study, we demonstrate that steroid phenotype is a key determinant of the adaptive immune response and clinical outcomes in ACC. LSP tumors exhibit significantly higher lymphocytic infiltration and greater repertoire diversity, reflecting a more immunogenic tumor microenvironment, despite the significant expression of immune evasion and exhaustion genes. These findings provide new insights into how the hormonal environment shapes the immunobiology of ACC, reveal possible mechanisms of immune escape in cortisol‐producing tumors, and highlight the prognostic relevance of immune repertoire characteristics. Our results support the integration of TCR/BCR profiling with steroid phenotyping to improve risk stratification and inform the design of precision immunotherapeutic strategies for ACC.

## 1 Introduction

Adrenocortical carcinoma (ACC) is a rare and highly aggressive neoplasm with a generally unfavorable prognosis and limited systemic therapeutic options. This highlights the urgent need for new treatment strategies and for prognostic and predictive biomarkers [1, 2, 3, 4, 5, 6, 7, 8]. The distinctiveness of ACC lies not only in its rarity but also in its complex pathophysiology, often marked by autonomous hypersecretion of cortisol, aldosterone, and androgens [9, 10]. Hormone excess ranges from subclinical states to overt syndromes (e.g., Cushing's syndrome), and both drive tumor pathogenesis and remodels the tumor microenvironment (TME), thereby shaping the host immune response. Previous studies have stratified ACC into distinct subtypes based on their steroidogenic profiles, such as the High Steroid Phenotype (HSP) and the Low Steroid Phenotype (LSP). These studies have revealed significant differences in clinical behavior and prognosis, with LSP being associated with more favorable outcomes [11, 12].

**FIGURE 1:**
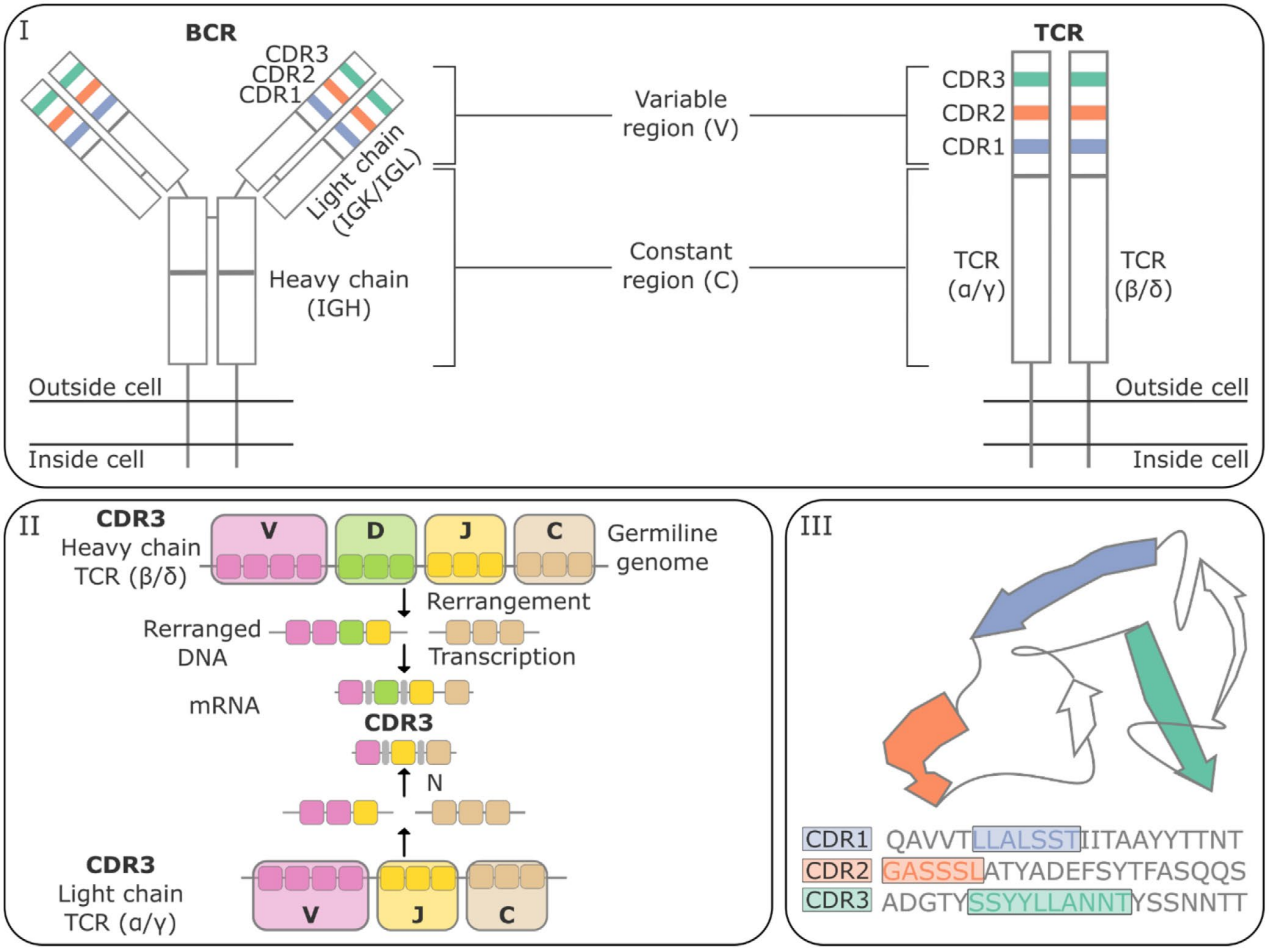
Structure of B‐cell receptors (BCRs) and T‐cell receptors (TCRs) and mechanisms of diversity generation. (I) Schematic representation of BCRs (comprising heavy and light chains) and TCRs (with α/β or γ/δ heterodimers). Both receptors are membrane‐anchored and contain constant (C) and cariable (V) regions. The Variable regions contain three Complementarity Determining Regions (CDR1, CDR2, and CDR3), which are primarily responsible for antigen binding. Specifically, the BCR heavy chain is encoded by the IGH gene, whereas the light chain is encoded by either the IGK (kappa) or IGL (lambda) gene. A functional BCR on the B‐cell surface consists of one IGH chain paired with either an IGK or IGL chain. (II) Illustration of the V (D) J somatic recombination process. Variable (V), Diversity (D, present in BCR heavy chains and TCR β/δ chains), and Joining (J) gene segments are randomly selected and rearranged at the DNA level in developing B and T lymphocytes. This process generates extensive diversity of the variable region. Imprecise joining of these segments, potentially involving the addition or deletion of nucleotides (N‐junctions), generates the hypervariable CDR3 region. (III) Simplified model of the receptor variable region, depicting the loops formed by CDR sequences projecting into the antigen‐binding interface. The highly diverse CDR3 sequence is unique to each B‐ or T‐lymphocyte clonotype, defining its antigen specificity, and is central to immune repertoire analysis for inferring immune response specificity and diversity.

The adaptive immune response, mediated primarily by T and B lymphocytes, is central to cancer surveillance and eradication. These lymphocytes' ability to recognize tumor antigens depends on their surface receptors: the B‐cell receptor (BCR) and the T‐cell receptor (TCR) [13]. The vast diversity of these receptors is generated by a somatic recombination process known as V(D)J recombination, which occurs during lymphocyte development in the bone marrow and thymus. This process involves the random assembly of variable (V), diversity (D), and joining (J) gene segments, along with junctional diversification through nucleotide insertions and deletions, resulting in the generation of the complementarity‐determining region 3 (CDR3) (Figure 1). The CDR3 is the most variable and functionally critical region of the receptor, primarily responsible for antigen‐binding specificity [14]. The uniqueness of the CDR3 sequence defines a clonotype, representing a population of lymphocytes with the same antigenic specificity. Analysis of the TCR and BCR repertoire, that is, the total set and frequency distribution of these clonotypes, allows for a deeper understanding of the nature, strength, and specificity of the adaptive immune response in different pathophysiological contexts, including cancer [13, 14, 15].

In the context of cancer, the immune repertoire provides valuable insights into host–tumor interactions. Tumors with high lymphocyte infiltration and a diverse repertoire, often referred to as “hot tumors”, are generally associated with a better prognosis and more favorable responses to immunotherapy, whereas “cold tumors” tend to be less responsive [16, 17, 18, 19, 20]. The quality, abundance, and diversity of TCR and BCR repertoires are critical indicators of the antitumor immune state [21, 22, 23, 24]. A comprehensive analysis of The Cancer Genome Atlas (TCGA), encompassing a wide range of tumors, identified six distinct immune subtypes, demonstrating the heterogeneity of the immune response across cancers and the importance of classifying tumors based on their immune profiles [25]. However, the TME is a complex and often immunosuppressive environment. It can shape and limit the effectiveness of the adaptive immune response, for example, through the action of immunosuppressive cytokines such as TGF‐β and regulatory cell populations that contribute to immune evasion [26, 27].

Classical inhibitory receptors such as PDCD1 (PD‐1, programmed cell death protein 1), CTLA4 (cytotoxic T‐lymphocyte associated protein 4), LAG3 (lymphocyte activation gene 3), HAVCR2 (TIM‐3), and TIGIT (T‐cell immunoreceptor with Ig and ITIM domains) suppress T‐cell receptor (TCR) signaling, leading to reduced cytokine production, proliferation, and cytotoxic activity [28, 29]. Beyond these immune checkpoints, additional genes contribute to immune evasion, T‐cell exhaustion, and impaired B‐ and T‐cell anti‐tumor function: TOX (thymocyte selection‐associated high mobility group box protein) is a master transcription factor that drives the epigenetic and transcriptional program of T‐cell exhaustion and promotes stable expression of inhibitory receptors (e.g., PD‐1, TIM‐3, TIGIT); NFIL3 (nuclear factor, interleukin 3 regulated; also called E4BP4) is a transcription factor that regulates NK cell development, CD8^+^ Tcell homeostasis, and circadian immune rhythms, contributing to immune evasion by reducing long‐term memory T‐cell responses and sustaining exhausted or terminally differentiated phenotypes; TCF7 (transcription factor 7; also called TCF‐1) maintains a pool of stem‐like progenitor exhausted T cells with proliferative capacity; NR3C1 (nuclear receptor subfamily 3 group C member 1; glucocorticoid receptor) encodes the receptor for cortisol and other glucocorticoids and, upon activation, suppresses proinflammatory cytokines (IL‐2, IFN‐γ, TNF‐α) while enhancing regulatory and suppressive programs. Finally, ENTPD1 (ectonucleoside triphosphate diphosphohydrolase 1; also called CD39) encodes an ectoenzyme expressed on exhausted T cells, regulatory T cells (Tregs), and some tumor cells, contributing to T‐cell suppression and metabolic immune evasion [30, 31].

Despite increasing research on the immune microenvironment in ACC, detailed characterization of TCR and BCR repertoires in relation to steroidogenic phenotype and its impact on prognosis remains at an early stage. Although it is known that steroid excess in HSP tumors can induce immunosuppression, leading to a “colder” immune profile and potential resistance to immunotherapy [12, 32, 33], how this immunosuppression is reflected in the diversity and clonal patterns of the adaptive immune repertoire and how it impacts clinical outcomes remain unexplored. Therefore, this study aimed to characterize and compare TCR and BCR repertoire profiles in ACC, stratified by high (HSP) and low (LSP) steroidogenic phenotypes, to elucidate their prognostic value and uncover immunological features shaped by the tumor hormonal microenvironment.

## 2 Material and Methods

### 2.1 Data Acquisition and Annotation

Raw RNA‐seq data (FASTQ/BAM) for 79 adrenocortical carcinoma (ACC) cases were obtained from the GDC Legacy Archive using the GDC Data Transfer Tool (https://gdc.cancer.gov/access‐data/gdc‐data‐transfer‐tool). Samples were annotated using the R/Bioconductor GenomicDataCommons v1.30.1 package [34], associating each sample with its TCGA barcode. Clinical data, including information for steroidogenic phenotypic classification and other relevant variables, were retrieved using the R/Bioconductor TCGAbiolinks v2.34.1 package [35]. Phenotypic annotations (high steroid phenotype, HSP; low steroid phenotype, LSP) followed published criteria [11, 12]. TPM‐normalized gene expression data on a log_2_ scale were downloaded from Xenabrowser (https://xenabrowser.net/datapages/).

### 2.2 Quality Control and RNA‐seq Pre‐Processing

Quality control and pre‐processing of RNA‐seq data were performed following the PreProcSEQ pipeline (detailed at https://bookdown.org/jean_souza/PreProcSEQ/). Read quality was assessed using FastQC (v0.12.0) [36], and aggregated results were summarized using MultiQC (v1.24) [37]. Next, Trimmomatic (v0.39) [38] was used to remove adapter sequences and trim low‐quality bases from the ends, according to the parameters recommended by the PreProcSEQ pipeline. The pre‐processed files were then used for subsequent immune repertoire reconstruction analyses.

### 2.3 B and T Cell Receptor Extraction

TRUST4 (v1.1.5) [39] was used to reconstruct BCR and TCR repertoires from the pre‐processed paired‐end RNA‐seq data. This approach identified and assembled sequences corresponding to the V(D)J regions of BCR heavy (IGH) and light (IGK, IGL) chains, as well as TCR alpha (TRA), beta (TRB), delta (TRD), and gamma (TRG) chains. The alignment and annotation processes followed the pipeline described in the official TRUST4 documentation (https://github.com/liulab‐dfci/TRUST4), using IMGT (International ImMunoGeneTics Information System) reference sequences for accurate identification of V, D, and J genes. Table S1 presents the average number of reads and clones reconstructed for the main BCR and TCR chains.

### 2.4 Definition and Identification of Immune Clones

A BCR clone was defined as a set of sequences sharing the same annotated V and J genes, possessing the same Complementarity Determining Region 3 (CDR3) length, and exhibiting at least 90% nucleotide sequence identity within the CDR3 region. Similarly, a TCR clone was defined as a set of sequences sharing the same V and J genes, the same CDR3 length, and a nucleotide identity in the CDR3 region of at least 95%. The analysis was restricted to productive sequences (in‐frame and without premature stop codons) containing complete CDR3 information, enabling subsequent diversity analyses. Due to the nature of RNA‐seq data from bulk tumor samples, which limits paired receptor chain analysis (heavy/light for BCR; alpha/beta or delta/gamma for TCR), each chain type was analyzed separately.

### 2.5 Comparative Analysis Between LSP and HSP Subgroups

ACC samples were classified into two subgroups based on their steroidal phenotype: HSP and LSP. This molecular classification followed the methodology established by Zheng et al. (2016) and adopted by Muzzi et al. (2021), which is based on on the expression profiles of key genes in the steroidogenesis pathway. The aim of this stratification was to compare the characteristics of the adaptive immune repertoire (BCR and TCR) between tumors with distinct molecular and hormonal profiles.

### 2.6 Correlation Analysis Between Repertoire Metrics and Lymphocyte Biomarkers

We assessed the association between receptor chain abundance, entropy and the expression of cell lineage marker genes. Using Spearman's correlation method, BCR chain metrics were correlated with the expression of B‐cell markers, whereas TCR chain metrics were correlated with T‐cell markers. The analysis considered the stratification of samples into low (LSP) and high (HSP) steroidogenic phenotypes.

### 2.7 Lymphocyte Infiltration and Diversity Analysis

To quantitatively assess lymphocyte infiltration and the general characteristics of the immune repertoire in each sample, the following metrics were calculated from TRUST4‐derived data: abundance (total number of identified TCR/BCR reads), normalized frequency (TCR/BCR reads per million total library reads,CPK), diversity (estimated using the Shannon index), and clonality (which quantifies unevenness in clonotype abundance, with higher values indicating dominance of a few clones). Additionally, to complement the estimation of immune cell infiltration using an orthogonal approach, the MCP‐counter method [40] and key cellular deconvolution metrics focused on the tumor microenvironment, implemented in the R package IOBR v. 0.99.0 [41], were used.

### 2.8 Association Analysis

Spearman correlations were calculated to assess monotonic associations between selected repertoire metrics (abundance and entropy per chain) and clinicomolecular variables. Analyses were performed across the entire cohort, with data also stratified into LSP and HSP groups. Correlations with a *p* value < 0.05 were considered nominally significant and visualized in heatmaps. Abundance and entropy were also evaluated in relation to tumor staging using the Mann–Whitney test. *p* values below 0.05 were considered significant.

### 2.9 Selection of Checkpoint Inhibitors and T‐Cell Exhaustion Genes

We selected a panel of genes representing key regulators of T‐cell exhaustion and adaptive immune resistance in the tumor microenvironment [30, 31]. To explore the functional relevance of these evasion genes, we analyzed their expression in relation to the abundance and diversity of BCR and TCR repertoires, as well as the inferred cellular composition of immune infiltrates. Cellular deconvolution was performed using algorithms implemented in the R package IOBR (v.0.99.0) [41]. In addition, we specifically investigated whether the expression of these T‐cell inhibitory genes was modulated by the tumor steroidogenic profile, comparing tumors with a high steroid phenotype (HSP) to those with a low steroid phenotype (LSP). This approach allowed us to evaluate whether glucocorticoid activity may differentially influence immune evasion mechanisms within the tumor microenvironment.

### 2.10 Survival Analysis

Overall survival analysis was performed using a multivariate Cox proportional hazards model adjusted for pathological stage and steroid phenotype, along with the Kaplan–Meier method, with curve comparisons between groups performed using the log‐rank test. Stratifications considered the following factors: LSP/HSP phenotype and high versus low abundance of BCR and TCR chains, dichotomized by the median. Analyses and visualizations were generated using the R packages survival (v3.7.0) [42] and survminer (v0.5.0) [43].

### 2.11 General Statistical Analysis

Statistical analyses were conducted in the R environment v. 4.4.2. For comparisons of continuous variables between groups that did not meet normality assumptions (assessed using the Shapiro–Wilk test, where applicable), nonparametric tests were employed. These included the two‐sided Mann–Whitney *U* test [44, 45] for comparisons when only two groups were available, and the Kruskal–Wallis rank sum test [46] for comparisons among more than two groups, followed by Dunn's test [47] for multiple pairwise comparisons. A significance level of α = 0.05 was adopted, with Benjamini–Hochberg FDR correction [48] applied for multiple comparisons. For heatmap construction, the R/Bioconductor ComplexHeatmap (v2.22.0) package was used [49].

## 3 Results

### 3.1 Study Design and Cohort Characterization

**FIGURE 2:**
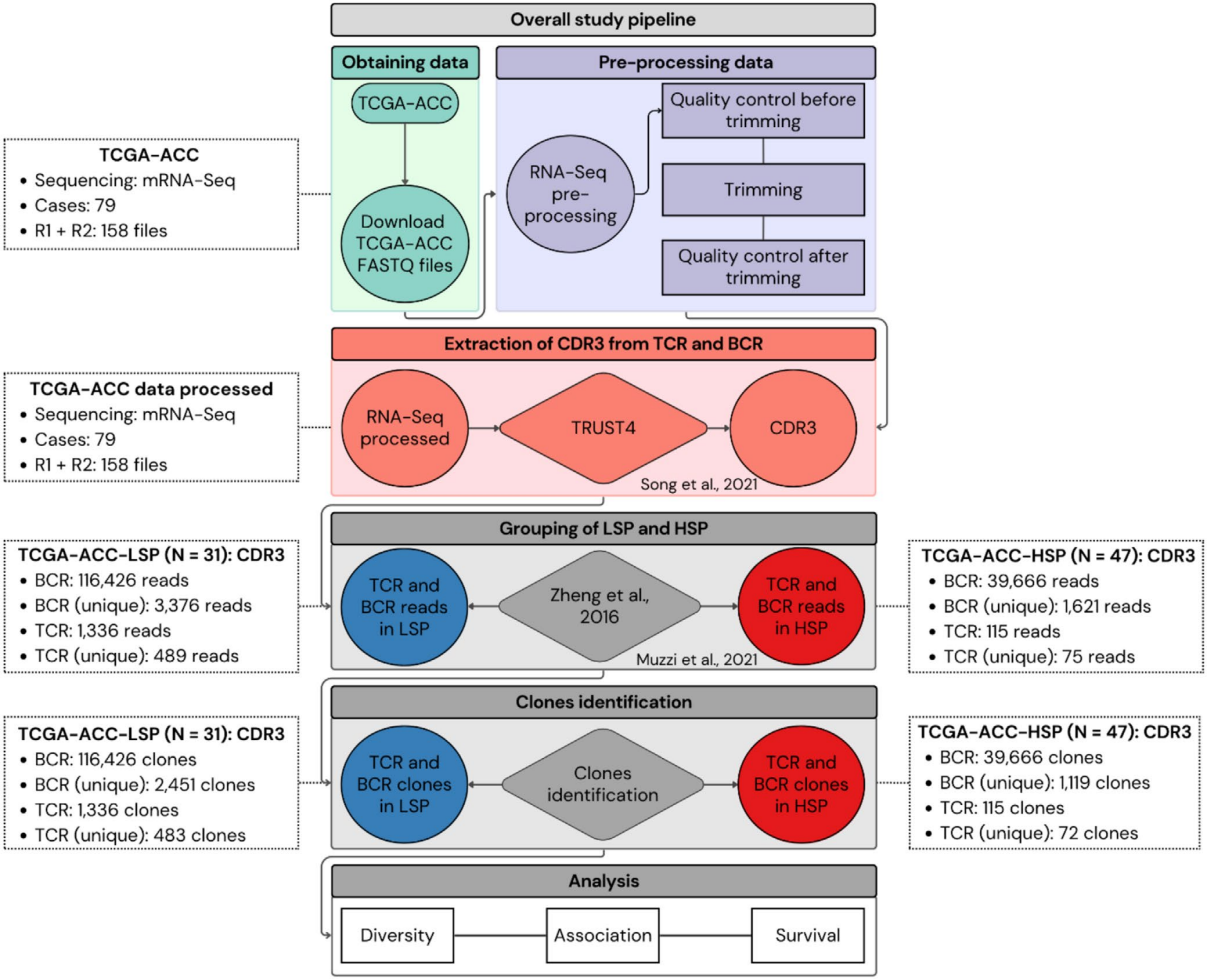
Overall study pipeline. This flowchart illustrates the analytical workflow of the present study, from obtaining ACC RNA‐seq data from TCGA to immune repertoire analyses. The main steps comprise: (1) Downloading of raw RNA‐Seq files; (2) Data preprocessing, including quality control and adapter removal; (3) Extraction of TCR and BCR CDR3 regions; (4) Clustering of samples into high steroid phenotype (HSP) and low steroid phenotype (LSP) subgroups, based on steroidogenic profiles; (5) Identification and quantification of TCR and BCR clones; and (6) Subsequent analyses of repertoire diversity, molecular associations, and survival. The side panels of the flowchart (TCGA‐ACC, processed TCGA‐ACC data, TCGA‐ACC‐LSP, TCGA‐ACC‐HSP) display, at each main step, the number of cases or reads and the total number of BCR and TCR clones (as well as unique clones) reconstructed for the LSP (*n* = 31) and HSP (*n* = 47) groups, providing a quantitative overview of data processing.

We analyzed B‐cell receptor (BCR) and T‐cell receptor (TCR) repertoires in 79 adrenocortical carcinoma (ACC) samples from The Cancer Genome Atlas (TCGA). Our goal was to characterize the immune infiltration and adaptive immune response in this tumor type. The complete study workflow is illustrated in Figure 2, covering RNA‐seq data acquisition and pre‐processing, BCR and TCR sequence extraction, and subsequent analyses of repertoire diversity, clonal expansion, associations with clinicopathological variables, and survival. Detailed results from RNA‐seq quality control and pre‐processing are presented in Figure S1, confirming the high quality of the input data for immune repertoire analyses.

The TCGA‐ACC cohort initially included 79 cases with available raw sequencing data. One case was excluded due to the absence of essential clinical information required for steroidogenic phenotype classification, resulting in a total of 78 ACC cases. The clinical and demographic characteristics of these patients, including age, sex, tumor stage, classification of excess cortisol, mineralocorticoids, sex steroids, and immune subtype classification, are summarized in Table 1. To enable comparative analyses based on the tumor hormonal profile, samples were stratified into HSP (*n* = 47) and LSP (*n* = 31). This stratification utilized clinical data obtained from TCGA and followed the methodology established by Zheng et al., (2016) and subsequently used by Muzzi et al., (2021).

**TABLE 1:** Clinical and demographic characteristics of adrenocortical carcinoma (ACC) cases according to steroidogenic phenotype.

	LSP (*N* = 31)	HSP (*N* = 47)
Total (dead)	31 (3)	47 (24)

	*N*/*N*_total_ (*N*_dead_/*N*)	*N*/*N*_total_ (*N*_dead_/*N*)
Male	15/31 (2/15)	16/47 (9/16)
Female	16/31 (1/16)	31/47 (15/31)
Mean age (male) ± SD	52.9 ± 11	46 ± 14.6
Mean age (female) ± SD	44.3 ± 16.1	46.8 ± 18.3
Tumor stage		
Stage I	6/31 (0/6)	3/47 (1/3)
Stage II	19/31 (1/19)	18/47 (6/18)
Stage III	4/31 (1/4)	12/47 (6/12)
Stage IV	2/31 (1/2)	12/47 (10/12)
Hormone excess cortisol	5/31 (1/5)	27/47 (14/27)
Mineralocorticoids	1/31 (0/1)	3/47 (1/3)
Sex steroids	8/31 (1/31)	20/47 (14/20)
Immune subtype		
C1	0/31 (0/0)	1/47 (1/1)
C2	1/31 (1/1)	0/47 (0/0)
C3	16/31 (0/16)	7/47 (3/7)
C4	12/31 (1/12)	37/47 (19/37)
C5	1/31 (0/1)	2/47 (1/2)
C6	1/31 (1/1)	0/47 (0/0)

*Note:* Clinical and demographic characteristics of adrenocortical carcinoma (ACC) cases (*N* = 78), stratified into HSP (*N* = 47) and LSP (*N* = 31). Values in parentheses indicate the number of deaths in each category, when applicable. The mean age is presented as mean ± standard deviation (SD) for each sex. Clinical data were obtained from the TCGA database. Samples were classified into LSP and HSP according to the methodology described by Zheng et al. (2016) [11] and subsequently applied by Muzzi et al. (2021) [12]. Immune subtypes were estimated Thorssson et al. (2018) [25].

### 3.2 Characterization and Abundance of B‐ and T‐Cell Repertoires

To characterize B‐ and T‐cell repertoires in the TCGA‐ACC cohort, the TRUST4 algorithm [39] was used to extract RNA‐seq sequences corresponding to BCR chains (IGH, IGK, and IGL) and TCR chains (TRA, TRB, TRD, and TRG). The choice of TRUST4 was based on its ability to reconstruct sequences spanning the complete V(D)J region, including complementarity‐determining regions (CDRs), which are essential for receptor specificity.

In total, 156,092 BCR reads and 1451 TCR reads were identified in the cohort (Figure 2). Initial analysis of these reads revealed a notable difference in abundance between the steroidogenic phenotypic groups: the LSP group (*n* = 31) presented 116,426 BCR reads (3376 unique reads) and 1336 TCR reads (489 unique reads). In contrast, the HSP group (*n* = 47) exhibited 39,666 BCR reads (1621 unique reads) and only 115 TCR reads (75 unique reads). These data consistently demonstrate a higher abundance of B‐ and T‐cell receptor transcripts in the LSP group compared to the HSP group (Figure 2, “TCGA‐ACC‐LSP” and “TCGA‐ACC‐HSP” panels). The number of reads identified for each BCR and TCR chain is detailed in Table S1.

The identified reads were subsequently grouped into immune clones. A clone was defined based on sharing the same V and J genes and a high nucleotide similarity in the CDR3 region (≥ 90% for BCRs and ≥ 95% for TCRs). This approach enables the estimation of cell populations derived from the same ancestor or with similar antigenic specificity. After clone identification and quantification (as summarized in Figure 2), the following were found: in the LSP group, a total of 2451 unique BCR clones and 483 unique TCR clones. In the HSP group, 1119 unique BCR clones and 72 unique TCR clones were identified.

These numbers of unique clones reinforce the observation of lower abundance in the HSP group and serve as a basis for assessing diversity within each group. The analysis with TRUST4 allowed the identification of BCR and/or TCR chain sequences in 68 of the 78 cases in the cohort (87.2%). Of these 68 cases with a detectable repertoire: 12 cases presented only BCR sequences, 8 cases contained only TCR sequences, and in 48 cases, sequences for both receptor types (BCR and TCR) were identified. In the remaining 10 cases (12.8%), no BCR or TCR sequences were detected above the method threshold with the available data, suggesting either absence or very low level of lymphocyte infiltration.

**FIGURE 3:**
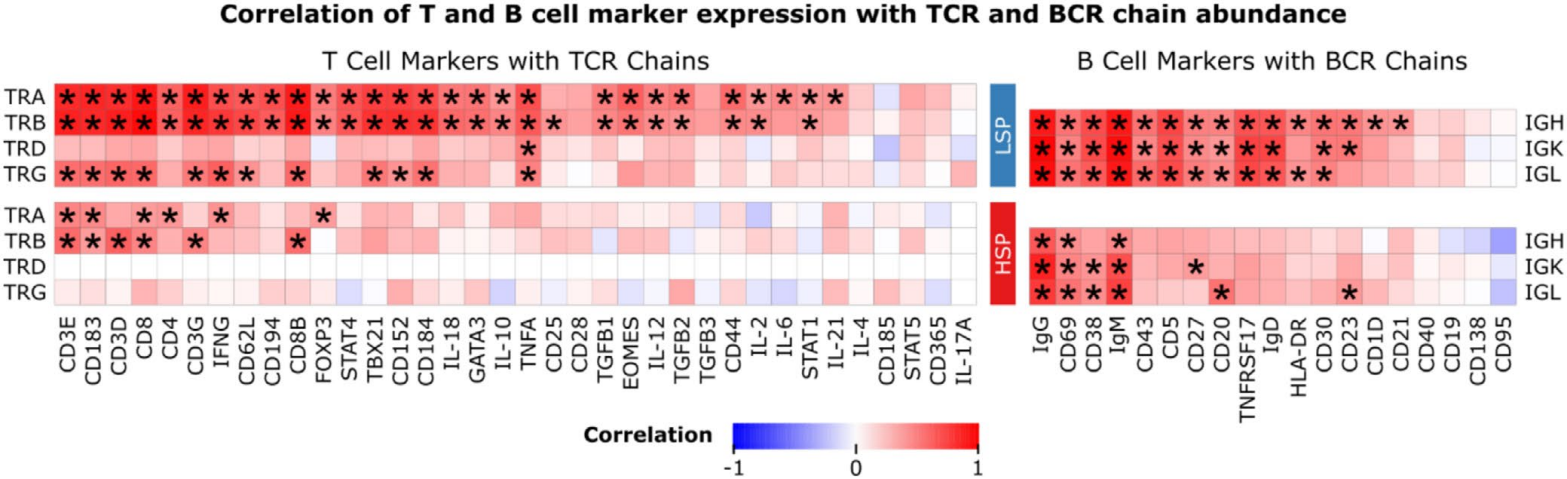
Correlation between immune receptor chain abundance and lineage‐specific lymphocyte markers in LSP and HSP adrenocortical carcinomas. Heatmaps represent Spearman correlation coefficients (ρ) between B‐cell receptor (BCR: IGH, IGK, IGL) and T‐cell receptor (TCR: TRA, TRB, TRD, TRG) chain abundance and their respective B‐ or T‐cell markers in LSP and HSP tumor subtypes. Marker expression and receptor chain abundance are measured as log_2_ (TPM + 1). Positive correlations are shown in red, negative correlations (ρ close to –1) in blue. The panels highlight differences in immune cell representation and transcriptional coordination between LSP and HSP tumor microenvironments. Cells marked with an ‘*’ indicate significant correlations (adjusted *p* < 0.05).

LSP tumors exhibited stronger and more widespread positive correlations between BCR chains (IGH, IGK, IGL) and classical B‐cell markers such as CD19, CD20, CD27, CD38, CD40, and immunoglobulin isotypes (IgM, IgG, IgD) (Figure 3). This suggests a more organized and functionally active B‐cell presence in tumors with low steroid production. In contrast, HSP tumors displayed an attenuated correlation pattern for most B‐cell markers, particularly weaker associations with CD19, CD27, and immunoglobulin isotypes, indicating a disrupted or suppressed B‐cell compartment in the context of elevated systemic glucocorticoid levels. For T cells, LSP tumors displayed robust correlations between TCR chains (TRA, TRB) and multiple effector markers, including CD3D/E/G, CD8, CD8B, IFNG, TBX21, and STAT4, indicating active T‐cell responses (Figure 3). HSP tumors, however, demonstrated an overall reduction in the strength of correlation between TCR and markers, especially involving CD8B, IFNG, and TBX21, consistent with glucocorticoid‐induced T‐cell exhaustion or suppression.

### 3.3 Differences in B‐ and T‐Cell Repertoire Infiltration and Diversity Between Steroidogenic Phenotypes

**FIGURE 4:**
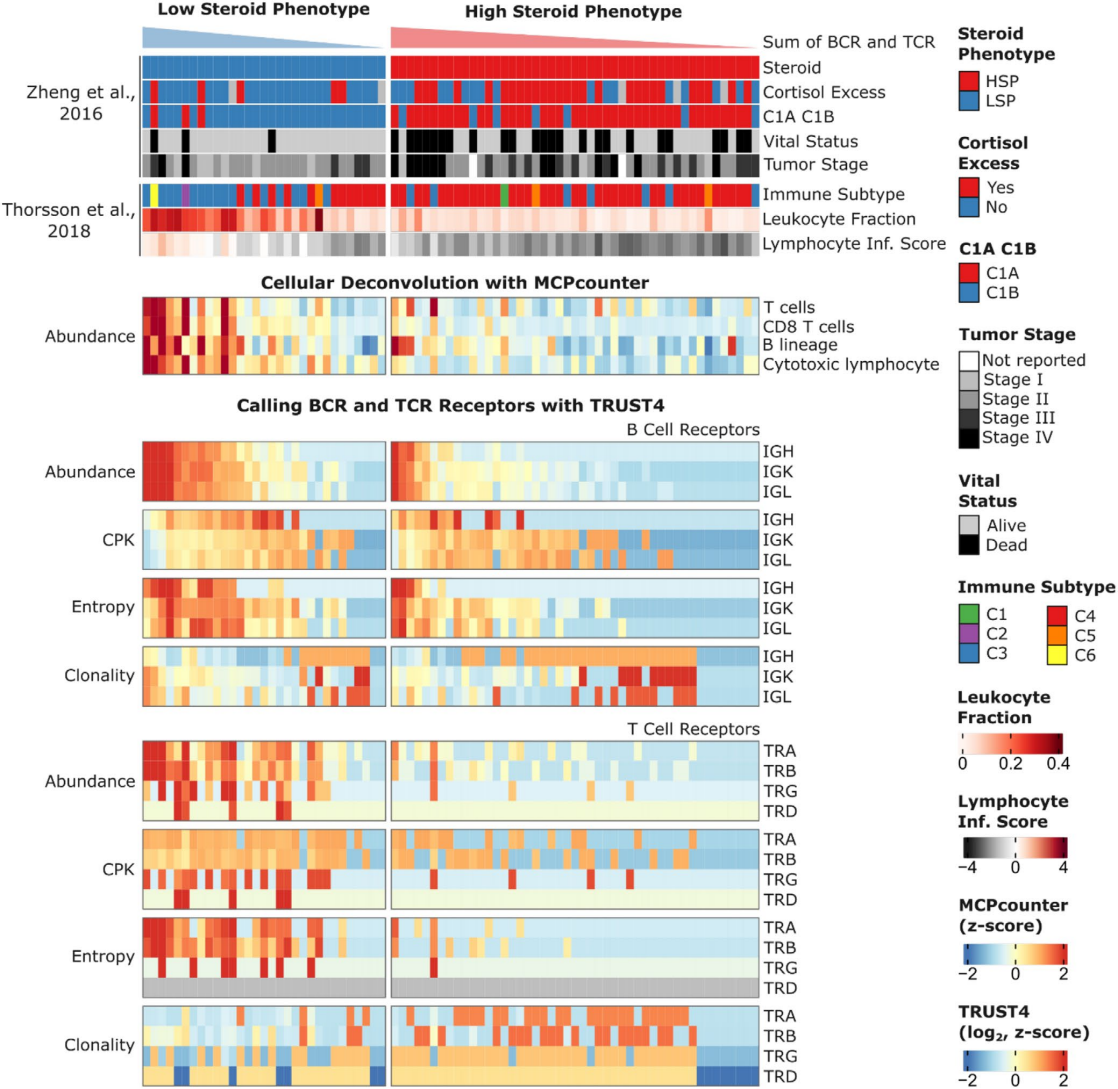
Characterization of the Immune Repertoire and Tumor Microenvironment Profile in ACC Stratified by Steroid Phenotype. This is a comparative heatmap of multiple immunological metrics and clinical characteristics in 78 ACC cases (TCGA). Columns represent individual cases, grouped and ordered by steroid phenotype (LSP on the left; HSP on the right) to facilitate pattern visualization. The top rows (colored bars) provide additional clinical and immunological metadata, including: steroid phenotype (LSP/HSP), cortisol excess, vital status, tumor stage [11], immune subtype, leukocyte fraction, and lymphocyte infiltration index [25]. The main body of the heatmap displays z‐score standardized values (color scale: Red indicates values above the mean, and blue indicates values below the mean) for the following metrics: cell deconvolution (MCP‐counter method), abundance, CPK, entropy, and clonality. Statistical differences between the LSP and HSP groups for repertoire metrics are detailed in Figures S3, S4 and S5.

To characterize the immune repertoires of the 78 ACC cases, stratified by their steroidogenic phenotype (LSP and HSP), we analyzed the main TCR and BCR repertoire metrics, visualized in the heatmap in Figure 4. The evaluated metrics included: abundance (total count of BCR or TCR reads, reflecting the frequency of receptor transcripts), CPK (Clones Per Kiloreads) (calculated as the number of unique clones per thousand total sequenced reads in the RNA‐seq library, serving as a measure of normalized richness), entropy (measure of repertoire diversity using the Shannon index, where higher values indicate greater diversity), and clonality (which quantifies the inequality in the frequency distribution of clones, with high values indicating that a few clones dominate the repertoire, implying lower diversity).

We observed that the difference in RNA‐seq library sizes did not significantly influence the abundance of TCR and BCR chains (Figure S2), eliminating the need for normalizing abundances based on library size. Detailed statistical differences for BCR and TCR metrics between the LSP and HSP groups are presented in Figures S3 and S4, respectively.

Comparative analyses between the LSP and HSP groups, visualized in Figure 4 and corroborated by statistical tests (Figures S3, S4 and S5), revealed striking differences in the immune repertoire. Tumors with the LSP phenotype consistently showed significantly higher abundance (total receptor reads) and entropy (diversity) for both receptor types (BCR and TCR) compared to HSP tumors. Only the TRD chain could not be compared due to the low number of detections, especially in the HSP group.

For BCR chains, CPK showed a significant difference only for the IGH chain, with greater clonal richness in LSP (Figure S3). For TCR chains, all, with the exception of TRB, exhibited a significantly higher CPK in the LSP group (Figure S4). Surprisingly, despite the differences in abundance and diversity, none of the BCR or TCR chains demonstrated a statistically significant difference in clonality between the LSP and HSP groups. However, the clone distribution in the LSP group tends to be more equitable, with less dominance from highly expanded clones, which is consistent with the greater clonal richness (CPK/abundance) observed for TCRs and comparable richness for BCRs between the groups.

The immune repertoire findings were corroborated by cell deconvolution estimates. Figure 4 includes the scores for different lymphocyte populations (MCP‐counter), showing greater T and B‐cell infiltration in LSP tumors (Figure S5). Collectively, these results indicate that LSP‐type ACC tumors are characterized by a more pronounced and diverse adaptive immune infiltration (B and T lymphocytes), associated with a potentially more immunogenic tumor microenvironment compared to HSP‐type tumors.

### 3.4 Associations Between B‐ and T‐Cell Repertoire and Clinicomolecular Characteristics of ACC

**FIGURE 5:**
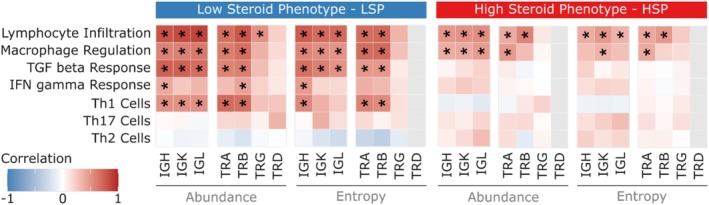
Spearman correlation heatmap between immune repertoire metrics and clinicomolecular characteristics in ACC, Stratified by Steroid Phenotype. Columns represent repertoire metrics (abundance and entropy for each BCR chain: IGH, IGK, IGL; and TCR chain: TRA, TRB, TRD, TRG), while rows represent clinicomolecular variables. Colors indicate the magnitude and direction of the correlation (blue = strong negative correlation [−1], red = strong positive correlation [+1], according to the scale). Asterisks (*) denote statistical significance (*p* < 0.05). SC stratification by steroid phenotype (LSP and HSP) is indicated in the top bars. Clinicomolecular variables and cell infiltration estimates were obtained from TCGA genomic and expression data analyses. The LSP/HSP classification followed the methodology by Zheng et al. (2016). This heatmap highlights distinct patterns of association between the immune repertoire and the tumor microenvironment in LSP and HSP groups, with the LSP phenotype demonstrating more robust associations with indicators of active immune response.

To investigate the relationships between immune repertoire features of the tumor microenvironment and ACC biology, Spearman correlations were calculated between the abundance and entropy of BCR and TCR chains and various clinicomolecular variables. The variables analyzed included: lymphocyte infiltration, macrophage regulation, TGF‐β response, IFN‐γ response, and Th1, Th17, and Th2 cell signatures as defined by Thorsson et al. (2018). These correlations were evaluated separately for the LSP and HSP groups. The complete results are presented in the heatmap in Figure 5.

Correlation analysis (Figure 5) revealed distinct patterns between the LSP and HSP subgroups. A positive correlation between the abundance and diversity of BCR and TCR chains and lymphocyte infiltration were consistently observed, being more prominent in the LSP group. This is an expected observation and corroborates previous findings (Figure 4). However, some correlations involving the TRG and TRD chains did not reach statistical significance, likely due to the reduced number of cases with detection of these chains, as previously observed. The correlation profile with macrophage regulation showed stronger interactions between TCR and BCR abundance/diversity in the LSP group, indicating potentially more efficient and coordinated immunological activity in this group.

The TGF‐β response showed a positive correlation with BCR and TCR abundance and diversity in the LSP group, an association absent or less pronounced in HSP. Similarly, the IFN‐γ response showed a positive correlation with the abundance and diversity of IGH and TRB chains in LSP but was absent in HSP. These findings suggest a more robust link between the presence of lymphocytes and the activation of pro‐inflammatory and antitumor immune pathways in LSP‐type tumors. Furthermore, a strong correlation was observed between the presence of Th1 cells and BCR and TCR abundance/diversity in LSP, but not in HSP, reinforcing the indication of a more active and directed T‐cell‐mediated immune response in the LSP phenotype.

Although several correlations were statistically significant, their magnitudes were moderate. It is important to emphasize that Spearman correlations only assess monotonic associations and do not allow for inferring cause‐and‐effect relationships.

### 3.5 Comparative Analysis of Immune Repertoire Abundance and Diversity Across Tumor Stages and Steroidogenic Phenotypes

**FIGURE 6:**
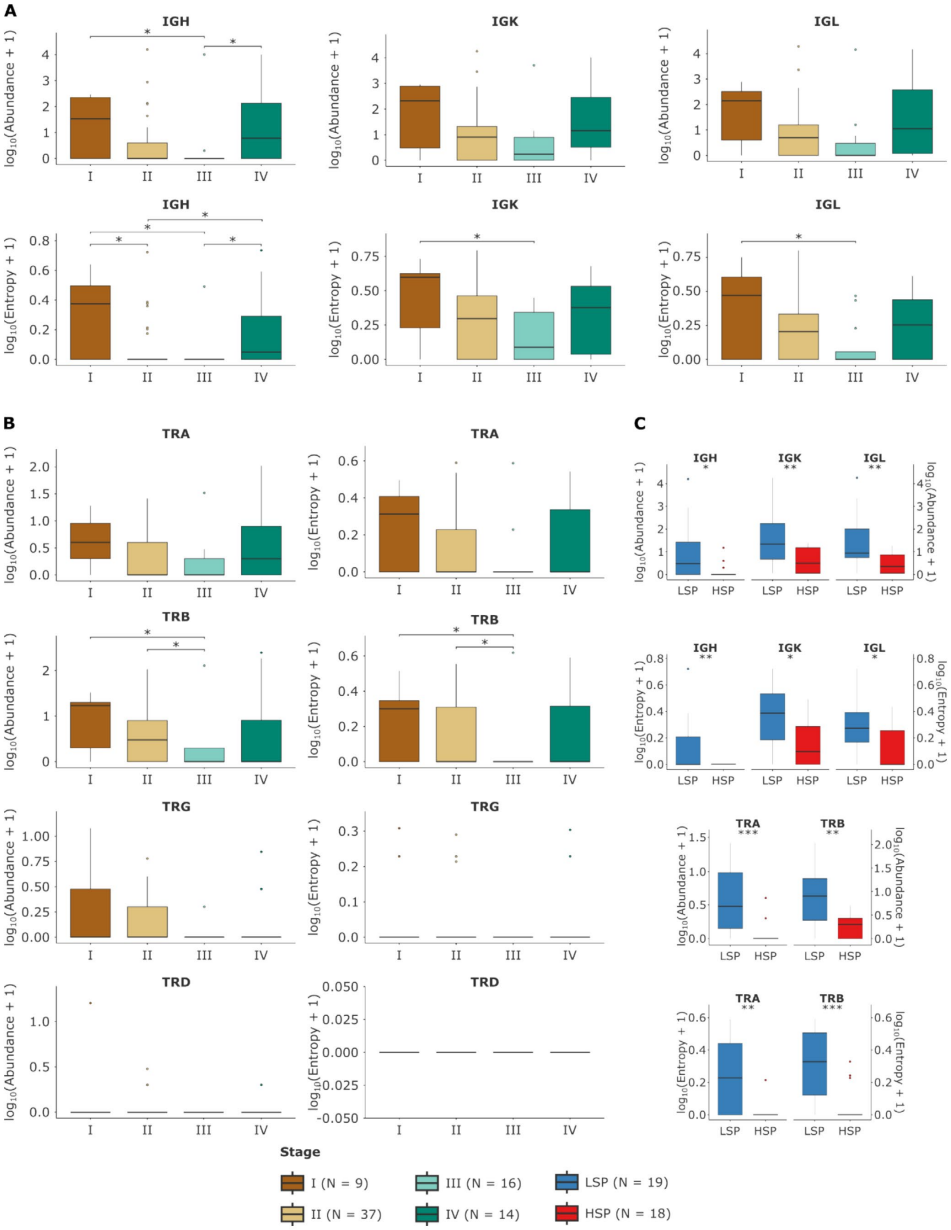
Comparison of BCR and TCR Chain Abundance and Diversity between Tumor Stages. Boxplots illustrate the comparison of BCR and TCR chain abundance and diversity between tumor stages (A and B) and steroidogenic phenotypes (C). For graphical representation, metric values were transformed to a log_2_(value +1) scale for normalization. The padj value (corrected for multiple comparisons via Benjamini–Hochberg FDR) is presented for each comparison. The Kruskal–Wallis test was used for comparisons between more than two groups (A and B), Dunn's test was used for pairwise multiple comparisons (A and B), and the Mann–Whitney test for comparisons between two groups (C).

Figure 6 examines the distribution of abundance (read counts) and diversity (Shannon entropy) of BCR chains (IGH, IGK, IGL) and TCR chains (TRA, TRB, TRD, and TRG) in relation to tumor staging and steroidogenic phenotype in ACC. This analysis was performed for 76 cases with complete staging information (Stage I, *n* = 9; Stage II, *n* = 37; Stage III, *n* = 16; and Stage IV, *n* = 14).

We evaluated chain abundance and diversity across different tumor stages individually (Stages I‐IV; BCR chains in Figure 6A and TCR chains in Figure 6B). For abundance, considerable variability was observed, with only a few statistically significant differences: for the IGH chain, between Stages I and III, and between III and IV (Figure 6A); and for the TRB chain, between Stages I and III, and between II and III (Figure 6B). Regarding diversity (entropy), all three BCR chains showed significant differences, with the IGH chain demonstrating more distinctions between stages (Figure 6A). For TCR chains, only the TRB chain showed a significant difference in diversity between the same stages that had differences in abundance (I and III, II and III; Figure 6B). However, it is important to note that the different tumor stages contain a mix of cases from both LSP and HSP groups, which may mask or confound the interpretation of direct associations with staging.

To obtain a more direct and robust comparison between steroidogenic phenotypes, minimizing the influence of tumor staging, we focused our analysis on a subgroup of 37 stage II cases. This subgroup was chosen because it included a comparable number of LSP (*n* = 19) and HSP (*n* = 18) cases (Figure 6C). Within this tumor‐stage‐homogeneous group, the differences between the LSP and HSP phenotypes became prominent and consistent. The LSP group demonstrated significantly higher levels of abundance and diversity than the HSP group for all BCR and TCR chains, with the exception of TRG and TRD chains, for which there was a low number of detected cases.

These results demonstrate that, when tumor staging is controlled (at least in Stage II, where comparison was possible), the greater abundance and diversity of BCR and TCR transcripts associated with the LSP phenotype are robust and highly significant characteristics. This strongly reinforces previous findings (Figures 4 and 5) indicating greater infiltration and adaptive immune activity in LSP tumors compared to HSP, regardless of tumor stage.

### 3.6 Analysis of Immune Evasion and Expression of Exhaustion Markers

**FIGURE 7:**
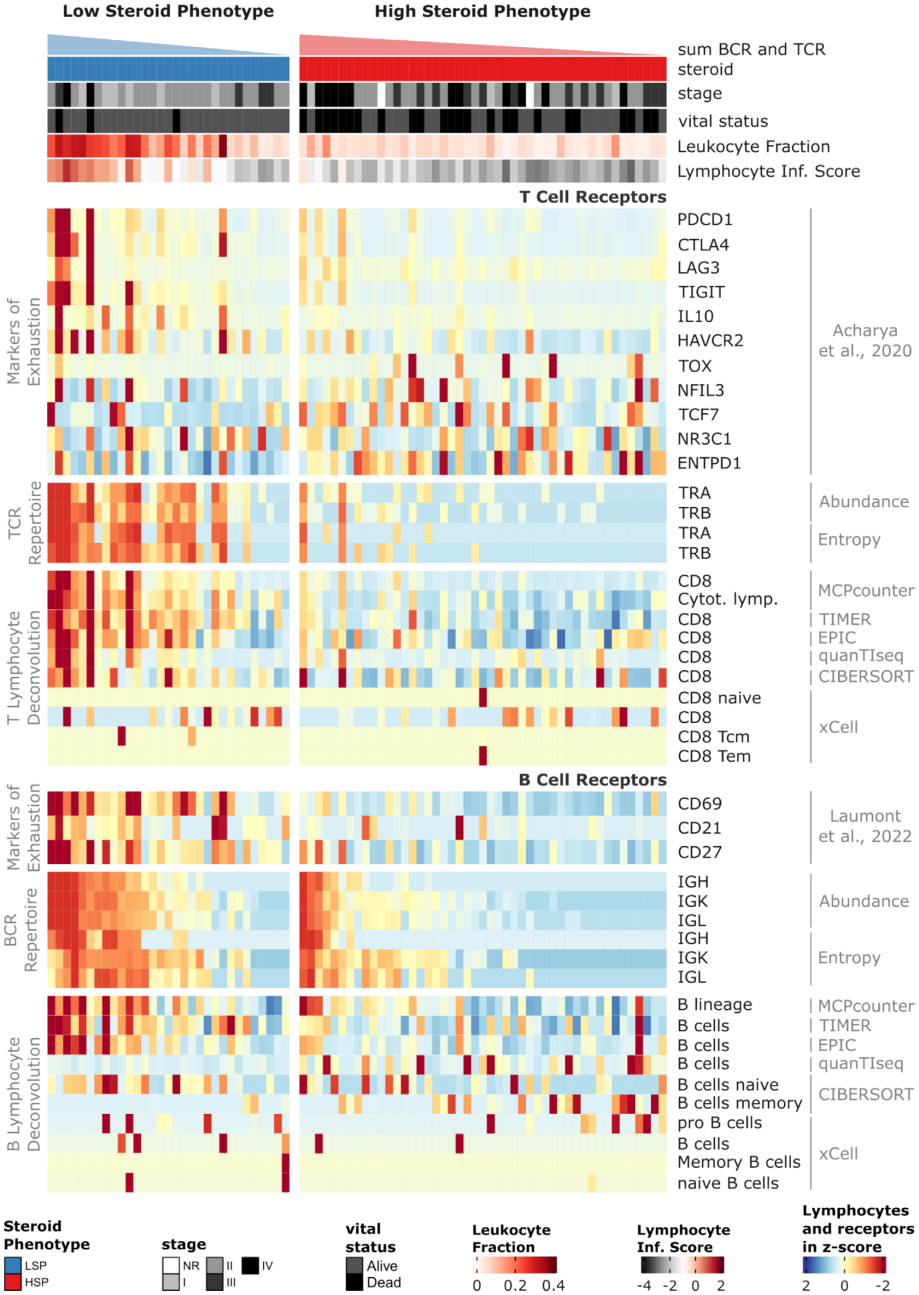
Characterization of T‐ and B‐cell exhaustion markers in the tumor microenvironment profile in ACC stratified by steroid phenotype. This is a comparative heatmap of gene expression of T‐ and B‐cell exhaustion markers with TCR and BCR abundance and diversity, and B‐ and T‐cell presence (cell deconvolution methods). Columns represent individual cases, grouped and ordered by steroid profile (LSP, on the left; HSP, on the right). Values in the heatmap were standardized by z‐score (color scale: Red indicates values above the mean, blue indicates values below the mean). The top rows provide additional metadata, including: steroid phenotype, tumor stage, vital status, leukocyte fraction, and infiltrating lymphocyte score. Statistical differences between the LSP and HSP groups for T‐ and B‐cell exhaustion markers are detailed in Figure S6.

To investigate the mechanisms of immune evasion that could explain the differences in the adaptive response between the steroid phenotypes, we evaluated the expression of canonical markers of T and B lymphocyte exhaustion directly in the heat map consolidating the cohort data (Figure 7 and Figure S6). This analysis sought to determine whether tumors with the HSP exhibit a molecular profile consistent with an immunosuppressive microenvironment. However, we found some overexpressed genes in LSP suggesting that chronic antigen stimulation and IFN‐driven signaling typically induce checkpoint receptors (PD‐1, LAG‐3, TIM‐3, TIGIT) as a negative‐feedback brake. The overexpression in LSP seems to be consistent with a robust antitumor response that's being actively restrained (adaptive immune resistance). The analysis revealed a clear pattern of overexpression of multiple immune checkpoints in the LSP group compared to the HSP group. Specifically for T‐cell exhaustion markers, a markedly elevated expression of PDCD1 (encoding PD‐1 protein; *p* < 0.001), TIGIT (p < 0.001), IL10 (*p* = 0.0048), and HAVCR2 (encoding TIM‐3; *p* = 0.00124) was observed in LSP tumors. In LSP tumors, TOX, NFIL3, TCF7, and ENTPD1 are expressed in a pattern consistent with chronic T‐cell stimulation: TOX and NFIL3 reflect progression toward exhaustion, ENTPD1/CD39 mediates metabolic suppression, and TCF7 highlights a progenitor subset that retains proliferative capacity. Together, these suggest that the immune system is highly active but constrained by exhaustion programs. In contrast, HSP tumors appear to rely more on cortisol/NR3C1‐mediated suppression, limiting T‐cell activation at earlier stages and thus showing weaker induction of these exhaustion‐associated transcripts.

Regarding tumor‐infiltrating B cells (TIL‐Bs), the expression of CD69 (*p* < 0.001), CD21 (*p* < 0.001), and CD27 (*p* < 0.001) markers differed between LSP and HSP (Figure 7 and Figure S6), demonstrating that LSP cases exhibit higher expression of these markers. Statistical comparisons supporting these expression differences between the LSP and HSP groups are presented in detail in Figure S6. This molecular signature suggests that infiltrating T and B cells in these tumors, although scarce, may be in an exhausted state, limiting their ability to mount an effective antitumor response.

The abundance and diversity of TCR and BCR chains, as well as cellular deconvolution focusing on CD8 and B cells using the main algorithms in this type of analysis, were presented together with the expressions of TIL and TIL‐B exhaustion markers in order to better understand the relationship of such markers with receptors and cell types (Figure 7).

### 3.7 Global Survival Analysis and the Prognostic Value of the Immune Repertoire

**FIGURE 8:**
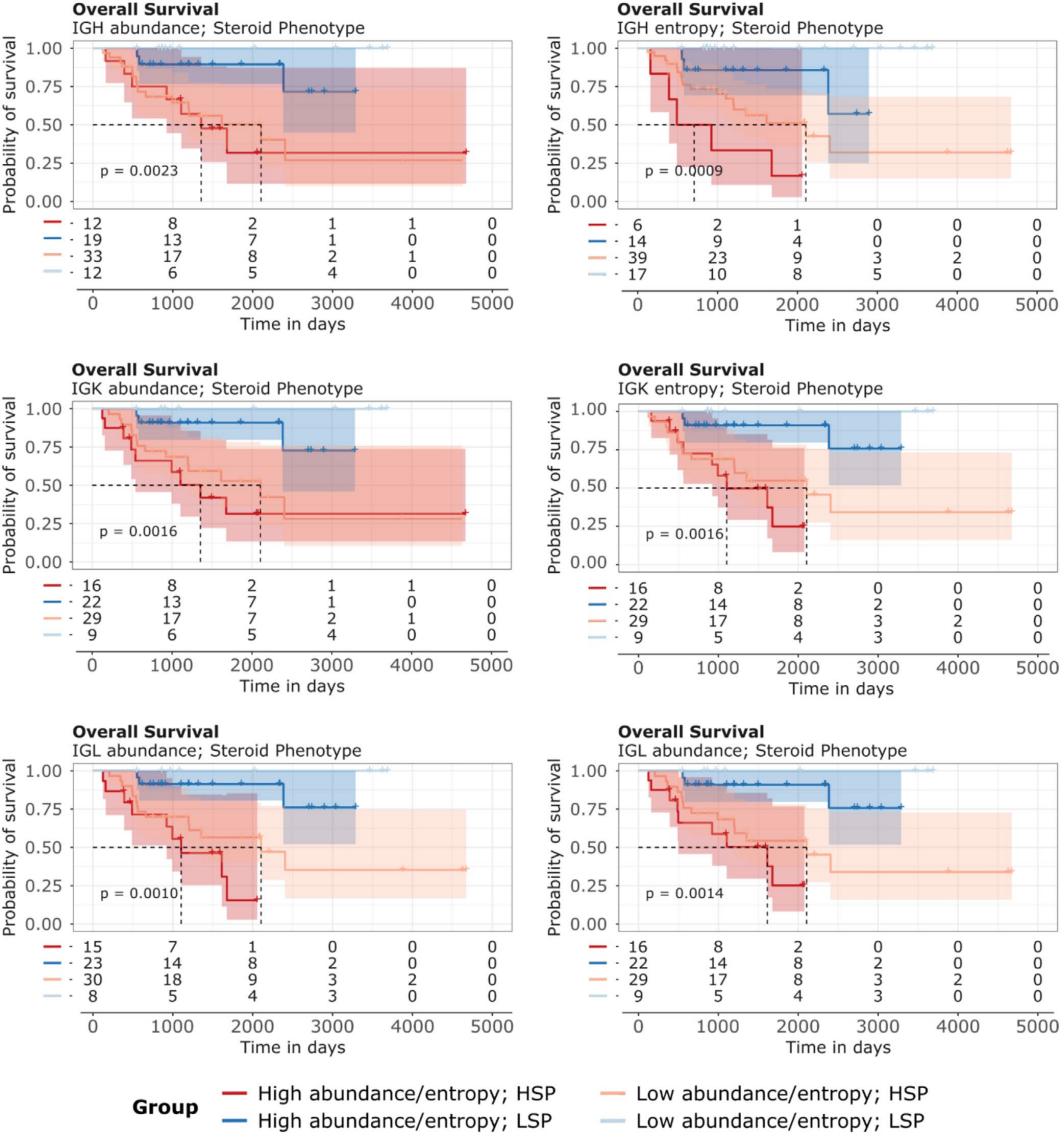
Analysis of overall survival in ACC associated with steroid phenotype and BCR repertoire characteristics. Kaplan–Meier curves for overall survival (OS) of ACC cases. Survival curve stratified by steroid phenotype and BCR chain abundance/entropy. BCR chain abundance is represented in the left panels, while their entropy is represented in the right panels. For each chain and phenotype, patients were dichotomized into “Above the Median” (High) and “Below the Median” (Low) groups based on the median read counts or entropy. *p* values (log‐rank test) indicate statistical differences between the compared curves. The number of cases at risk at specific time intervals is represented below each graph.

**FIGURE 9:**
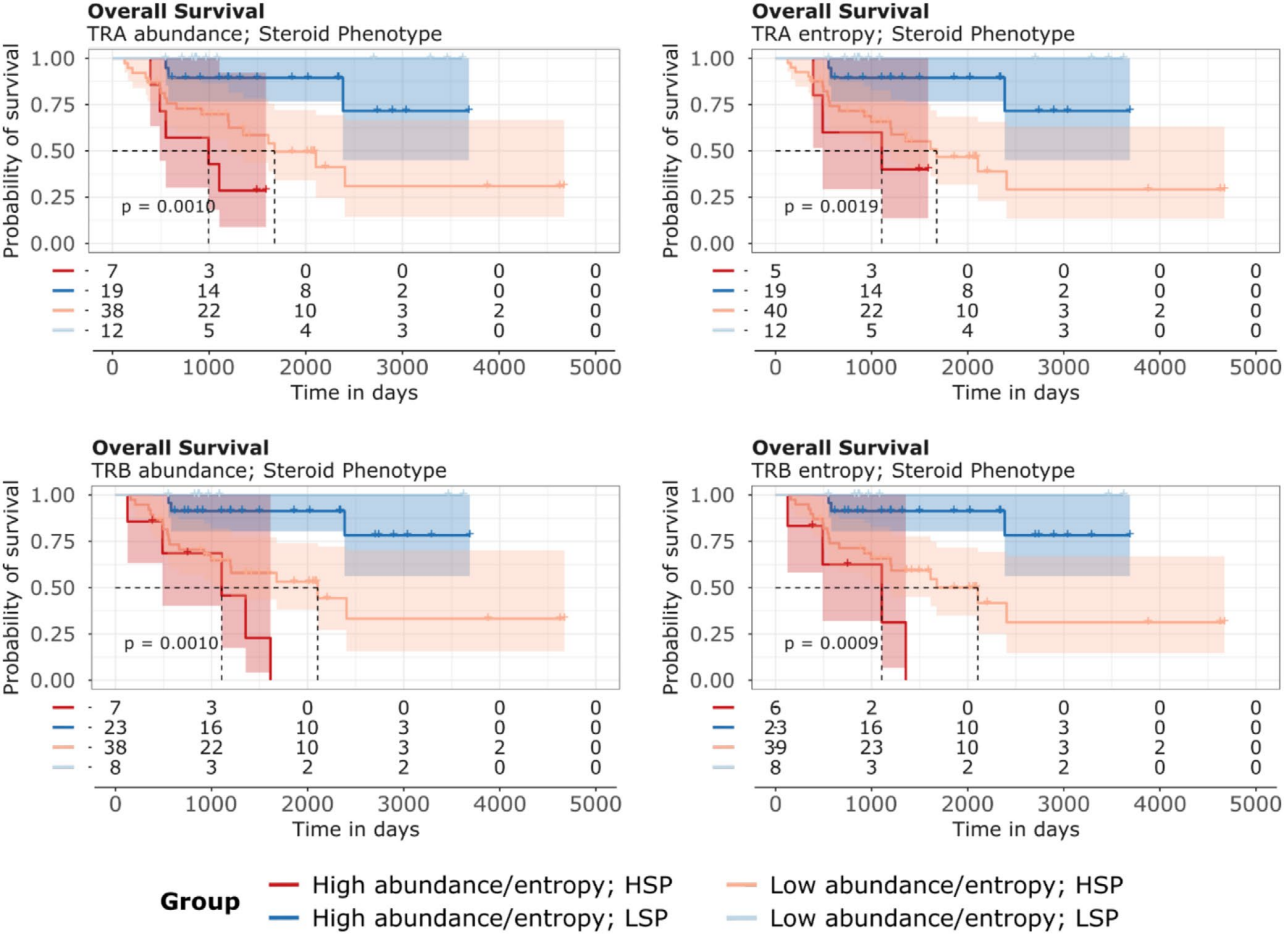
Kaplan–Meier curves for overall survival (OS) of ACC cases associated with steroid phenotype and TCR repertoire characteristics. Survival curves are stratified by steroid phenotype and TCR chain abundance/entropy. TCR chain abundance is represented in the left panels, while TCR chain entropy is represented in the right panels. For each chain and phenotype, patients were dichotomized into “Above the Median” (High) and “Below the Median” (Low) groups based on the median read counts or entropy. *p* values (log‐rank test) indicate statistical differences between the compared curves. The number of cases at risk at specific time intervals is represented below each graph.

We assessed the relationship between immune repertoire abundance and diversity and overall survival (OS) in ACC cases using Kaplan–Meier curves (Figures 8 and 9). As evidenced by previous studies [12], cases with the LSP phenotype exhibit significantly superior survival compared to those with the HSP phenotype. In this work, we examined whether the abundance and diversity of BCR (Figure 8) and TCR (Figure 9) chains could have prognostic value within the LSP and HSP groups. The detailed results from Kaplan–Meier analyses demonstrated that metrics of BCR and TCR chain abundance and diversity have prognostic value, with patterns varying between LSP and HSP phenotypes.

**FIGURE 10:**
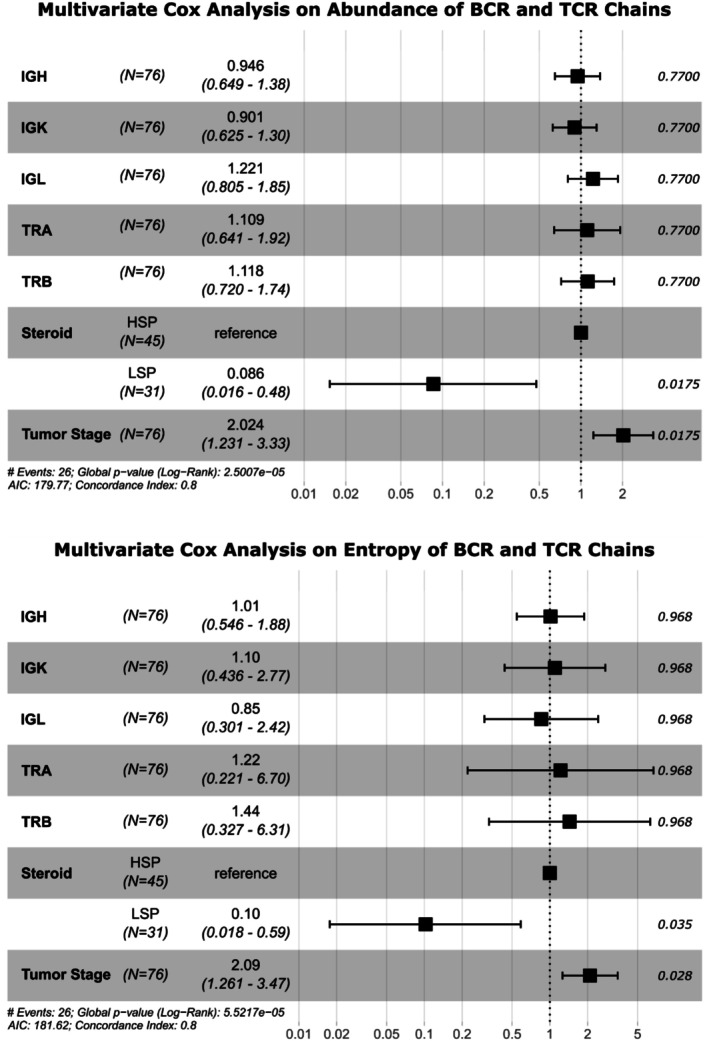
Forest plot representing Cox multivariate analysis for abundance and entropy of BCR and TCR chains adjusted for steroidogenic phenotype and tumor stage. The X‐axis represents the risk of the event (death), values above contribute to the event occurring, values below contribute to the event not occurring, and values equal to one indicate that the variable has no influence on the occurrence of the event. The *p* values are presented for each variable, as well as the number of cases, the hazard ratio, and the confidence interval.

In all scenarios, the variable that appears to have the most significant impact on prognosis is the steroidal phenotype profile, not the abundance or entropy of the BCR and TCR chains. Both BCR chains (Figure 8) and TCR chains (Figure 9) show a trend toward better prognosis in LSP cases compared to HSP. The results of the multivariate Cox analysis considering the effect of the abundance or entropy of the BCR and TCR chains adjusted for steroidogenic phenotype and tumor stage corroborate these findings (Figure 10), as do the results of the multivariate Cox analysis of each chain adjusted for steroidal phenotype and tumor stage (Table 2). In both cases, the risk effect is significantly affected by the steroidogenic phenotype and tumor stage, indicating that LSP cases have a lower risk of death compared to HSP cases, and cases with more advanced stages have a higher risk of death.

**TABLE 2:** Multivariate Cox analysis for abundance and entropy of BCR and TCR chains in ACC cases.

	HR	Lower 0.95	Upper 0.95	*p*	*p* ^adj^
**Abundance**					
IGH	1.07	0.96	1.21	0.234	0.842
IGK	1.08	0.95	1.21	0.236	0.842
IGL	1.09	0.98	1.23	0.126	0.842
TRA	1.32	0.93	1.87	0.117	0.842
TRB	1.23	0.95	1.59	0.115	0.842
IGH:IGK	1.00	0.96	1.03	0.885	1.000
IGH:IGL	1.00	0.97	1.03	1.000	1.000
TRA:TRB	1.10	0.98	1.23	0.098	0.842
**Entropy**					
IGH	1.10	0.72	1.69	0.647	0.961
IGK	1.13	0.77	1.66	0.524	0.961
IGL	1.11	0.73	1.70	0.632	0.961
TRA	1.65	0.79	3.45	0.180	0.842
TRB	1.70	0.85	3.30	0.139	0.842
IGH:IGK	0.83	0.54	1.28	0.399	0.961
IGH:IGL	0.92	0.68	1.24	0.574	0.961
TRA:TRB	1.01	0.45	2.28	0.976	1.000
**Abundance: HSP**					
IGH	1.00	0.90	1.20	0.597	0.961
IGK	1.10	0.92	1.20	0.460	0.961
IGL	1.10	0.95	1.20	0.263	0.842
TRA	1.10	0.70	1.70	0.691	0.961
TRB	1.10	0.74	1.50	0.769	0.984
IGH:IGK	1.00	0.96	1.04	0.957	1.000
IGH:IGL	1.00	0.96	1.04	0.958	1.000
TRA:TRB	1.08	0.78	1.52	0.635	0.961
**Entropy: HSP**					
IGH	1.10	0.66	1.80	0.764	0.984
IGK	1.10	0.74	1.70	0.562	0.961
IGL	1.20	0.74	2.00	0.457	0.961
TRA	1.30	0.49	3.50	0.578	0.961
TRB	1.60	0.71	3.70	0.254	0.842
IGH:IGK	1.05	0.62	1.78	0.862	1.000
IGH:IGL	1.03	0.69	1.52	0.898	1.000
TRA:TRB	0.59	0.05	6.42	0.665	0.961

*Note:* TCR chains (TRA and TRB); BCR chains (IGH, IGK, and IGL). Interaction of IGH with IGK (IGH:IGK), interaction of IGH with IGL (IGH:IGL), interaction of TRA with TRB (TRA:TRB). Lower 95%, start of the confidence interval; Upper 95%, end of the confidence interval.

Abbreviations: HR, Hazard Ratio; *p* value, *p* ^ajd^, adjusted *p* value.

## 4 Discussion

In this study, we employed an immunoinformatic approach to analyze the TCR and BCR repertoires in TCGA ACC cohort, stratified by their steroidogenic phenotype. Our findings demonstrate that the steroidogenic phenotype is intrinsically linked to the adaptive immune response in ACC, with LSP tumors exhibiting a more robust and active immune profile characterized by greater infiltration and diversity which correlate with a better prognosis. This more immunogenic/antigen‐rich TME in LSP may induce adaptive up‐regulation of checkpoints (PD‐1, CTLA‐4, LAG‐3, TIGIT, TIM‐3), not found not found in steroid‐suppressed activation (HSP).

We first observed that LSP tumors exhibit significantly greater abundance and clonal diversity of BCR and TCR transcripts compared to HSP tumors. This increased lymphocyte infiltration in the LSP tumor microenvironment was corroborated by cell deconvolution analyses using MCP‐counter and other methods (Figure 4, and Supplementary Figures S5 and S6), which indicated a greater presence of diverse B‐ and T‐cell populations. These findings align with growing evidence that adaptive immune cell infiltration is a critical prognostic biomarker across multiple cancer types, including ACC [16, 17, 18, 19, 50, 51, 52, 53, 54, 55, 56].

Analysis of Spearman correlations between immune receptor chains and lineage‐specific markers revealed distinct immunological profiles between LSP and HSP adrenocortical tumors. In LSP tumors, BCR chains (IGH, IGK, IGL) showed strong positive correlations with key B‐cell markers such as CD19, CD20, CD27, CD38, CD40, and immunoglobulin isotypes (IgM, IgG, IgD), consistent with a differentiated and transcriptionally coordinated B‐cell compartment. In contrast, HSP tumors exhibited marked attenuation of these correlations, consistent with glucocorticoid‐mediated suppression of B‐cell receptor signaling and immunoglobulin transcription. Similarly, TCR chains (TRA, TRB, TRD, TRG) showed strong correlations with T‐cell activation and effector markers, including CD3D, CD8B, IFNG, TBX21, and STAT4, in LSP tumors, suggesting an active or Th1‐skewed cytotoxic response. However, these relationships were globally weakened in ACC‐HSP tumors, consistent with cortisol‐induced inhibition of T‐bet and IFN‐γ expression and impaired CD8^+^ T‐cell effector function [57].

The immunosuppressive effects of excess glucocorticoids, characteristic of HSP tumors, likely underlie their sparse immune infiltration and unfavorable prognosis. Cortisol inhibits T cell proliferation, induces lymphocyte apoptosis, and impairs antigen presentation [32, 33]. These hormonal effects may drive an immune‐excluded or “cold” tumor phenotype, contributing to resistance to immunotherapy. The scarcity of immune cells in the HSP tumor microenvironment, therefore, suggests an immune escape mechanism mediated by steroidal hypersecretion, which may partly explain the unfavorable prognosis associated with this subtype [12, 32, 33], given that cortisol‐secreting ACC tumors exhibit an immunosuppressive profile, potentially accounting for their poor prognosis and resistance to immunotherapy [33].

Beyond infiltration, repertoire diversity further distinguished LSP tumors, as indicated by the significantly higher entropy and CPK indices for most TCR and BCR chains (Figure 4, Supplementary Figures S3 and S4). Recent studies have shown that greater TCR and BCR repertoire diversity is frequently associated with a more effective immune responses and better prognosis in cancer cases, reflecting the immune system's ability to recognize and respond to a broader range of antigens [18, 22, 23]. This characteristic suggests that LSP tumors are more “immunogenic” or allow for a more complete and varied adaptive immune response.

Correlation analyses between repertoire metrics and tumor microenvironment characteristics (Figure 5) reinforce the immunological distinction between LSP and HSP phenotypes. It was expected that the abundance and diversity of BCR and TCR would correlate positively with total lymphocyte infiltration, and this association was particularly prominent in the LSP group, highlighting the greater presence of active adaptive immune cells. Similarly, the positive correlation between repertoire and macrophage regulation in LSP tumors suggests a tumor microenvironment where immune cells, including macrophages, are more likely to be in a proinflammatory and antitumor state, in contrast to the polarization toward immunosuppressive (M2) profiles frequently observed in aggressive tumors [58].

Differential correlations with cytokine pathways are especially informative. The positive association of the LSP repertoire with IFN‐γ response and the presence of Th1 cells indicates an activated immune microenvironment directed towards a Th1‐type response, which is classically associated with tumor control and immunotherapy efficacy [59]. In contrast, the absence of these correlations in the HSP tumors suggests a less responsive or suppressed environment to these crucial pathways. Unexpectedly, the LSP immune‐repertoire score correlated positively with a TGF‐β–response signature, despite TGF‐β's canonical immunosuppressive role in promoting T‐cell exhaustion and M2‐like macrophage polarization [26, 27]. However, its role is complex and context‐dependent; for example, high TGF‐β signaling can nullify the favorable prognostic value of Th1 immune responses in some cancer types [60, 61]. It is possible that, in an already proinflammatory microenvironment such as LSP, TGF‐β is involved in regulatory feedback mechanisms, or that its presence is indicative of an active immune response that attempts, but not always succeeds, in overcoming its suppressive effects [62, 63].

We also evaluated the impact of tumor stage on the abundance and diversity of the immune repertoire. Although we observed specific associations between BCR and TCR abundance and diversity metrics and individual tumor stages (Figure 6A,B), interpretation of these relationships was challenging due to the heterogeneity of LSP and HSP tumors within each stage. This mix of steroidogenic profiles, with their distinct immunological characteristics, can mask or confound the true influence of pure staging on the immune repertoire [12, 51, 56].

To overcome this limitation and isolate the effect of the steroidogenic phenotype, we performed a focused analysis in a more homogeneous subgroup of 37 patients with Stage II tumors, which presented a balanced distribution between LSP and HSP phenotypes (Figure 6C). Within this stage‐controlled group, differences in immune repertoire abundance and diversity between LSP and HSP tumors became notably prominent and statistically significant for almost all BCR and TCR chains. This finding is particularly important as it robustly demonstrates that the steroidogenic phenotype is a major determinant of the adaptive immune response in ACC, capable of modulating lymphocyte infiltration and diversity more significantly than tumor stage itself, or at least independently of the complexity associated with advanced staging. This suggests that the hormonal environment establishes an intrinsic “immune signature” that persists even when other prognostic factors, such as disease stage, are controlled. This level of control is crucial for uncovering underlying biological factors of the immune response in complex cancers such as ACC [12, 32, 56, 64].

Comparative analysis of the immune microenvironment between LSP and HSP phenotypes reveals two distinct and sophisticated mechanisms of tumor immune evasion. Steroid signaling (NR3C1) may reprogram T cells toward a quiescent or regulatory state, rather than a classic program that co‐expresses multiple checkpoints. One mechanism seems to be independent of the steroid influence, where chronic antigen stimulation and IFN‐driven signaling typically induce checkpoint receptors (PD‐1, LAG‐3, TIM‐3, TIGIT) as a negative‐feedback brake. Overexpression of multiple inhibitory receptors, such as PD‐1 (PDCD1), CTLA4, TIGIT, and TIM‐3 (HAVCR2), is a hallmark of T‐cell exhaustion that arises after chronic antigenic stimulation, such as that which occurs in immunogenic tumors [30, 65, 66]. This suggests that, in the LSP microenvironment, an adaptive immune response is indeed mounted but is subsequently suppressed by mechanisms of adaptive tumor resistance. The coexistence of strong infiltration with markers of exhaustion indicates that these tumors represent an archetypal “inflamed but ineffective” microenvironment, making them theoretically ideal candidates for immune checkpoint blockade therapies that aim to reverse this exhausted state.

In contrast, the second mechanism, involves the HSP phenotype, which resembles an immunological desert or “pauci‐immune” state, where the primary evasion strategy is not suppression of a preexisting response, but rather exclusion or prevention of lymphocyte infiltration. However, our findings of elevated ENTPD1 (CD39) and TCF7 expression in this group add a crucial layer of complexity. High ENTPD1 expression points to an actively immunosuppressive microenvironment through the adenosine pathway, where hydrolysis of extracellular ATP generates adenosine, a potent suppressor of T cell and other immune cell function [67]. This suppression mechanism does not depend on cell–cell interactions (such as PD‐1/PD‐L1), but rather on metabolic modulation of the microenvironment. Simultaneously, the increased expression of TCF7, a transcription factor essential for the maintenance of stem‐like/progenitor exhausted T cells [68, 69], in HSP tumors may indicate that the few T lymphocytes that manage to infiltrate are maintained in an undifferentiated state, unable to proliferate and generate a robust effector response.

Analysis of the tumor‐infiltrating B lymphocyte (TIL‐B) compartment in LSP tumors reveals a complex molecular signature, but one more indicative of a functional response than exhaustion (Figure 7; Figure S6). Importantly, because we used bulk RNA‐seq data, we cannot confirm the co‐expression of these markers in individual B cells. However, the collective gene signature, activation (CD69), memory (CD27), and germinal center support (CD21), converges on a biologically plausible hypothesis: LSP tumors are characterized by the formation of Tertiary Lymphoid Structures (TLSs). These structures, which function as ectopic germinal centers, are known to orchestrate sophisticated antitumor responses and are often associated with improved prognosis and response to immunotherapy [70]. Therefore, our data suggest that the B‐cell compartment in LSP tumors is highly dynamic, mature, and likely organized into functional TLEs, actively contributing to the antitumor immune response, although this response may ultimately be contained by T‐cell exhaustion.

Survival analysis reveals one of the central findings of our study: The steroidogenic phenotype emerges as the most dominant prognostic factor in adrenocortical carcinoma, surpassing the independent predictive value of immune repertoire metrics. Kaplan–Meier curves (Figures 8 and 9) consistently demonstrate that LSP tumors have significantly superior overall survival to those with the HSP. Although univariate analyses could suggest a benefit from a richer or more diverse repertoire, stratification by phenotype already visually indicates that the main separation in the survival curves occurs between the LSP and HSP groups, rather than between high and low abundance or entropy within each group.

The robustness of this conclusion is supported by multivariate Cox analysis (Figure 10 and Table 2). When adjusting the model for the steroid phenotype and tumor stage, both known prognostic factors in ACC, the apparent effect of TCR and BCR chain abundance and entropy becomes statistically insignificant. Hazard ratios (HRs) for all repertoire metrics approach 1.0, with confidence intervals crossing this value and nonsignificant *p* values. In contrast, the LSP phenotype maintains a massive and independent protective effect, with a hazard ratio of approximately 0.1, indicating an approximately 90% reduction in the risk of death compared to the HSP phenotype. This demonstrates that the steroid phenotype does not act as a simple confounder, but rather as the primary determinant of clinical outcome.

We propose a biological model in which immune receptor repertoire characteristics are not an independent driver of survival, but rather a reflection of the underlying tumor microenvironment, which is fundamentally shaped by the steroidogenic phenotype. As discussed, the LSP phenotype corresponds to an immunologically “hot” microenvironment, characterized by high lymphocyte infiltration, the presence of TLS, and an active, albeit partially exhausted, immune response. Consequently, a greater abundance and diversity of lymphocyte receptors is an intrinsic and expected characteristic of this “hot” environment. Conversely, the HSP phenotype represents a “cold” immunological desert, where low infiltration results, by definition, in a poorer repertoire. Therefore, multivariate Cox analysis suggests that, although a rich repertoire is a biomarker of the LSP phenotype, it is the phenotype itself, with all its complex machinery of immune suppression or permissiveness, that holds the true prognostic power.

While studies in other cancers, such as melanoma and pancreatic cancer, have directly associated greater TCR and BCR repertoire diversity with improved survival [18, 71], our results suggest that in ACC, the hormonal and metabolic context imposed by steroidogenesis is such a powerful “master regulator” that it overshadows the repertoire's secondary effects. In short, it is not enough to have an army of lymphocytes (high abundance) with many different specialties (high diversity); the decisive factor is whether this army operates in a permissive environment (LSP) or a hostile and suppressive terrain (HSP). This finding has important implications for the design of future clinical trials in ACC, suggesting that stratifying patients based on steroidogenesis phenotype may be more critical than assessing the repertoire alone.

Despite the novel insights provided by this study, several limitations should be acknowledged. First, the analysis relied on bulk RNA sequencing data from the TCGA‐ACC cohort, which does not allow resolution of immune repertoire features at the single‐cell level, nor can it distinguish between tumor‐infiltrating lymphocytes and peripheral immune cells. Second, the inference of BCR and TCR clonotypes from bulk RNA‐seq, while informative, is inherently limited in sensitivity and accuracy compared to dedicated immune repertoire sequencing technologies (e.g., AIRR‐seq, scTCR/BCR‐seq). Third, the relatively small sample size of ACC cases, particularly after stratification by steroidogenic phenotype, may reduce the statistical power to detect associations or generalize findings across heterogeneous tumor subgroups. Finally, while the classification into LSP and HSP phenotypes was based on validated gene expression criteria, the dynamic nature of steroid hormone secretion in vivo, particularly cortisol, may introduce variability not fully captured in transcriptomic snapshots. Future studies integrating single‐cell multiomics, spatial profiling, and clinical response data will be essential to validate and extend these findings.

## 5 Conclusions

This study establishes that the biology of steroidogenesis fundamentally modulates the architecture and functional state of the immune microenvironment in adrenocortical carcinoma (ACC), emerging as a strong prognostic determinant than immune receptor repertoire metrics alone. We identify two distinct immunological endotypes: The low steroidogenic phenotype, which corresponds to an immunologically permeable (“hot”) tumor microenvironment characterized by high T‐ and B‐ lymphocyte density, clonal diversity, and the formation of tertiary lymphoid structures, whose antitumor efficacy is limited by the coexpression of multiple inhibitory receptors; and the high steroidogenic phenotype, which represents an immune‐excluded (“cold”) microenvironment defined by metabolic barriers and active suppression mechanisms. This dichotomy provides a framework for rational patient stratification, with direct translational implications for immunotherapy. Some LSP tumors exhibit features consistent with candidacy for T‐cell–augmenting therapies, most notably immune checkpoint blockade. Conversely, overcoming therapeutic resistance in HSP patients, and other LSP cases under significant subclinical cortisol levels, may require earlier inhibition of steroid synthesis (e.g., during immunotherapy). Thus, this work provides a mechanistic framework to guide the development of personalized and precision therapeutic approaches in ACC.

## Author Contributions

All authors contributed substantially to the design of the work. J.S.S.R. was responsible for data acquisition and analysis. M.A.A.C. and J.C.D.M. contributed to data acquisition and analysis methods. All authors contributed to data interpretation. J.S.S.R. and B.C.F. wrote the first draft of the manuscript. I.S.G. assisted in code review. E.L. and J.A.M.B. contributed to manuscript writing and review. All authors contributed to the article and approved the submitted version.

## Funding

This project was funded by Behring Foundation, Brazil, PRONON (2024‐76.591.569/0001‐30, 25000.029124/2021‐31), Brazil and the CNRS *IIPACT* International Research Network, France. BCF is recipient of a 1B CNPq Research Productivity Scholarship.

## Ethics Statement

The studies involving human participants were reviewed and approved by Pequeno Príncipe Ethics Committee (7.616.381). Written informed consent from the participants' legal guardian/next of kin was not required to participate in this study in accordance with the national legislation and the institutional requirements.

## Conflicts of Interest

The authors declare no conflicts of interest.

## Supporting information


**Table S1:** Contagens de Leituras e Clones do Repertório de BCR e TCR por Fenótipo de Esteroide.
**Figure S1:** RNA‐Seq Data Quality Control Metrics. This figure assesses the quality and integrity of RNA‐Seq reads before and after pre‐processing. Panels (A) and (B) display the average quality scores across the read length for all samples, indicating high base quality, before and after pre‐processing respectively. Panels (C; before pre‐processing) and (D; after pre‐processing) illustrate the low content of “N” bases (indeterminate bases) along the read length, reflecting good sequencing quality. Panels (E; before pre‐processing) and (F; after pre‐processing) indicate the percentage of adapter content along the read length; notably, panel (F) evidences that no significant adapter contamination (> 0.1%) was detected in any of the samples after the trimming step performed by the PreProcSEQ pipeline. These results confirm that the RNA‐Seq data possess the necessary quality for robust immune repertoire analyses.
**Figure S2:** Correlation Matrix Between Repertoire Abundance Metrics and RNA‐Seq Library Size. This is a heatmap displaying the Spearman correlation coefficients between abundance counts for each individual receptor chain (IGH, IGK, IGL, TRA, TRB, TRD, TRG), total BCR and TCR reads, the overall repertoire sum (Sum of TCR and BCR), and the total RNA‐Seq sequencing library size for each case. The intensity and color of the cells represent the Spearman's rho correlation coefficient, ranging from blue (strong negative correlation, −1) to red (strong positive correlation, +1), according to the indicated color scale. Cells marked with an “X” indicate nonsignificant correlations (*p* > 0.05). This analysis demonstrates the interrelationships among the abundances of different chains and, crucially, evaluates the dependence of repertoire metrics on the total sequencing depth (library size). It confirms that the abundance of BCR and TCR chains is not significantly affected by library size, as indicated by the low correlation coefficients in the first row (“RNA‐Seq library”).
**Figure S3:** Comparison of Immunological Repertoire Metrics for BCR Chains. Boxplots illustrate the comparison of Abundance, CPK (Clones per Kiloreads), Entropy (Shannon diversity), and Clonality metrics for B‐cell receptor (BCR) chains (IGH, IGK, and IGL) between the Low Steroid Phenotype (LSP, *N* = 31, in blue) and High Steroid Phenotype (HSP, *N* = 47, in red) groups. For graphical representation, metric values were transformed to a log_2_ (value+1) scale for normalization. The padj value (corrected for multiple comparisons via Benjamini–Hochberg FDR) is presented for each comparison. The Mann–Whitney test was used for group comparisons. The results demonstrate that the LSP group exhibits higher abundance and diversity for all BCR chains, and higher CPK for IGH, with statistically significant differences (p.adj < 0.05) for IGH, IGK, and IGL Abundance, IGH CPK, and IGH and IGL Entropy. Clonality, however, shows no significant differences.
**Figure S4:** Comparison of Immunological Repertoire Metrics for TCR Chains. Boxplots illustrate the comparison of Abundance, CPK (Clones per Kiloreads), Entropy (Shannon diversity), and Clonality metrics for T‐cell receptor (TCR) chains (TRA, TRB, TRD, and TRG) between the Low Steroid Phenotype (LSP, *N* = 31, in blue) and High Steroid Phenotype (HSP, *N* = 47, in red) groups. For graphical representation, metric values were transformed to a log_2_ (value+1) scale for normalization. The padj value (corrected for multiple comparisons via Benjamini–Hochberg FDR) is presented for each comparison. The Mann–Whitney test was used for group comparisons. Entropy for TRD could not be calculated due to the low detection frequency of this chain, especially in the HSP group. The results indicate that the LSP group shows significantly higher Abundance, CPK, and Entropy for most TCR chains (p.adj < 0.05), with the notable exception of the TRB chain which did not show a significant difference for CPK, and TRD which had limited data. Clonality does not exhibit significant differences between the groups.
**Figure S5:** Comparison of MCP‐counter scores for T cells, CD8 cells, B cell lineage, and cytotoxic lymphocytes. Boxplots illustrate the comparison of T‐ and B‐cell deconvolution scores between the low steroid phenotype (LSP, *N* = 31, in blue) and high steroid phenotype (HSP, *N* = 47, in red) groups. For graphical representation, metric values were transformed to a log_2_ (value+1) scale for normalization. The *p* value is presented for each comparison. The Mann–Whitney test was used for group comparisons.
**Figure S6:** Comparison of MCP‐counter scores for T cells, CD8 cells, B‐cell lineage, and cytotoxic lymphocytes. Boxplots illustrate the comparison of T‐ and B‐cell deconvolution scores between the low steroid phenotype (LSP, *N* = 31, in blue) and high steroid phenotype (HSP, *N* = 47, in red) groups. For graphical representation, metric values were transformed to a log_2_ (value+1) scale for normalization. The *p* value is presented for each comparison. The Mann–Whitney test was used for group comparisons.

## Data Availability

The original contributions and presentations in the study are included in the GitHub repository (https://github.com/jean‐resende/BCR_and_TCR_repertoire_in_TCGA‐ACC). Additional questions can be directed to the corresponding author. All scripts, datasets, software, and algorithms used to generate results, figures, and tables for this study are available in the GitHub repository (https://github.com/jean‐resende/BCR_and_TCR_repertoire_in_TCGA‐ACC).
