# Supplementary material for: The Steroidal Profile Modulates Adaptive Immune Response and Prognosis in Adrenocortical Carcinoma: Analysis of TCR and BCR Repertoires
Source: Cancer Med. 2026 Jun 5;15(6):e71781. doi: 10.1002/cam4.71781 (PMC13241620; doi:10.1002/cam4.71781)
Supplement: Supplementary file 1 — Table S1: Contagens de Leituras e Clones do Repertório de BCR e TCR por Fenótipo de Esteroide. Figure S1: RNA‐Seq Data Quality Control Metrics. This figure assesses the quality and integrity of RNA‐Seq reads before and after pre‐processing. Panels (A) and (B) display the average quality scores across the read length for all samples, indicating high base quality, before and after pre‐processing respectively. Panels (C; before pre‐processing) and (D; after pre‐processing) illustrate the low content of “N” bases (indeterminate bases) along the read length, reflecting good sequencing quality. Panels (E; before pre‐processing) and (F; after pre‐processing) indicate the percentage of adapter content along the read length; notably, panel (F) evidences that no significant adapter contamination (> 0.1%) was detected in any of the samples after the trimming step performed by the PreProcSEQ pipeline. These results confirm that the RNA‐Seq data possess the necessary quality for robust immune repertoire analyses. Figure S2: Correlation Matrix Between Repertoire Abundance Metrics and RNA‐Seq Library Size. This is a heatmap displaying the Spearman correlation coefficients between abundance counts for each individual receptor chain (IGH, IGK, IGL, TRA, TRB, TRD, TRG), total BCR and TCR reads, the overall repertoire sum (Sum of TCR and BCR), and the total RNA‐Seq sequencing library size for each case. The intensity and color of the cells represent the Spearman's rho correlation coefficient, ranging from blue (strong negative correlation, −1) to red (strong positive correlation, +1), according to the indicated color scale. Cells marked with an “X” indicate nonsignificant correlations (p > 0.05). This analysis demonstrates the interrelationships among the abundances of different chains and, crucially, evaluates the dependence of repertoire metrics on the total sequencing depth (library size). It confirms that the abundance of BCR and TCR chains is not significantly affected by library [file CAM4-15-e71781-s001.docx]

# Suplementary Material

**Table S1 – Contagens de Leituras e Clones do Repertório de BCR e TCR por Fenótipo de Esteroide.**

| Chain | **HSP (N = 47) Median (Min - Max) [N de casos com recptor]** | **LSP (N = 31) Median (Min - Max) [N de casos com recptor]** |
| --- | --- | --- |
| **BCR reads**  IGH | 14 (1 – 10,188) [13] | 120 (1 – 15,913) [19] |
| IGK | 10 (1 – 10,382) [29] | 111 (1 – 18,096) [26] |
| IGL  **BCR clones** | 5.5 (1 – 14,735) [28] | 96.5 (1 – 19,269) [24] |
| IGH | 15.5 (1 – 10,178) [12] | 120 (1 – 15,912) [19] |
| IGK | 10 (1 – 10,382) [29] | 111 (1 – 18,096) [26] |
| IGL  **TCR reads**  TRA  TRB  TRD  TRG  **TCR clones**  TRA  TRB  TRD  TRG | 5.5 (1 – 14,735) [28]  1 (1 – 10) [15]  1 (1 – 30) [19]  0 (0 – 0) [0]  1 (1 – 2) [4]  1 (1 – 10) [15]  1 (1 – 30) [19]  0 (0 – 0) [0]  1 (1 – 2) [4] | 96.5 (1 – 19,269) [24]  7 (1 – 103) [25]  16 (1 – 246) [26]  1 (1 – 15) [5]  2 (1 – 11) [15]  7 (1 – 103) [25]  16 (1 – 246) [26]  1 (1 – 15) [5]  2 (1 – 11) [15] |

**TABLE S1** Read and Clone Counts for BCR and TCR Repertoires by Steroid Phenotype in Adrenocortical Carcinoma (ACC). This table provides a quantitative summary of read abundance and the number of identified clones for each B-cell receptor (BCR) chain (IGH, IGK, IGL) and T-cell receptor (TCR) chain (TRA, TRB, TRD, TRG). Data are stratified by High Steroid Phenotype (HSP, N = 47) and Low Steroid Phenotype (LSP, N = 31) groups. For each chain and phenotype, we present the median count, the observed minimum and maximum values (min – max), and the number of cases [N] within each group where the respective chain was detected. The definitions of reads and clones adhere to those detailed in the Materials and Methods section.


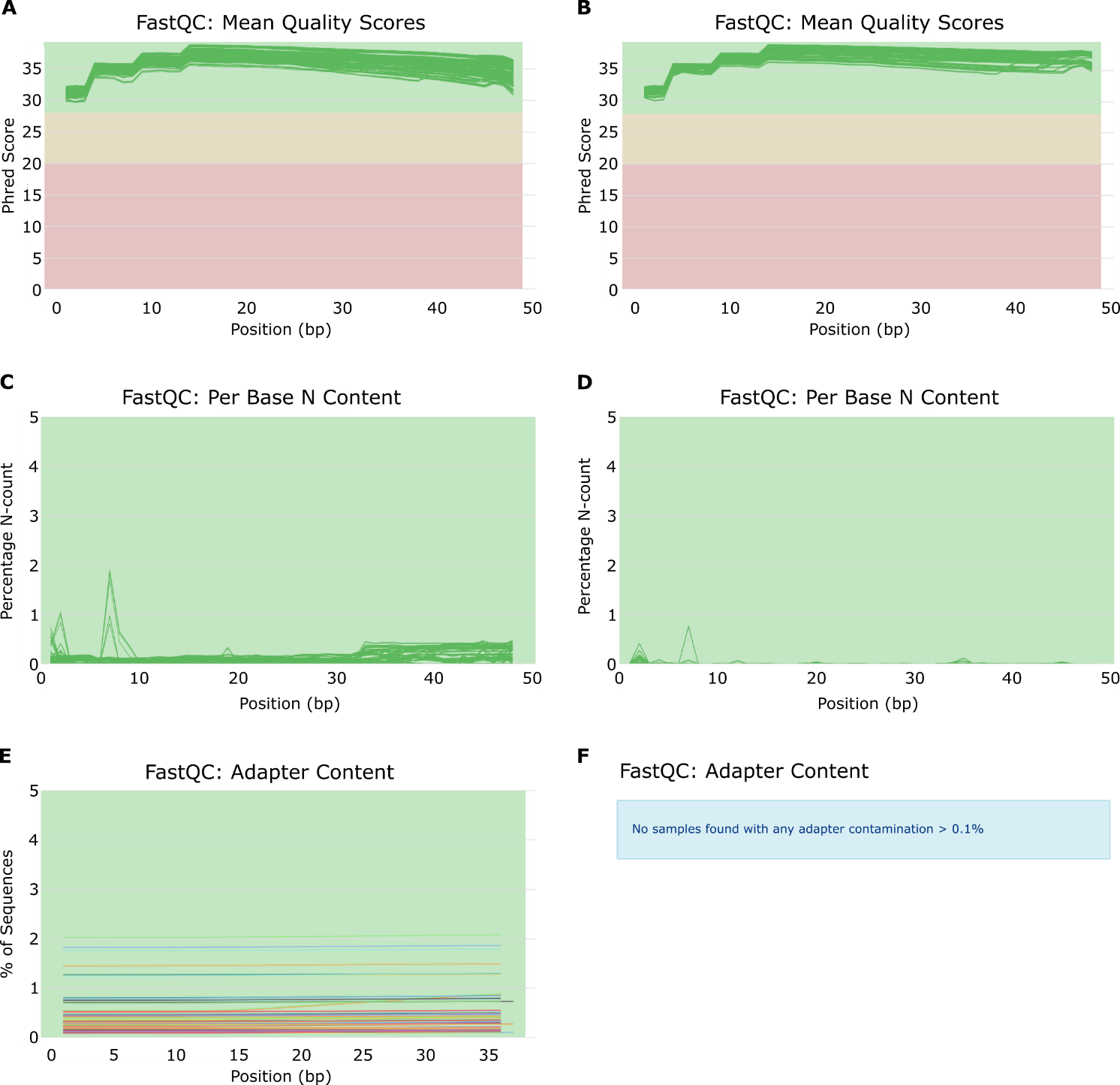


**FIGURE S1** RNA-Seq Data Quality Control Metrics. This figure assesses the quality and integrity of RNA-Seq reads before and after pre-processing. Panels (A) and (B) display the average quality scores across the read length for all samples, indicating high base quality, before and after pre-processing respectively. Panels (C; before pre-processing) and (D; after pre-processing) illustrate the low content of "N" bases (indeterminate bases) along the read length, reflecting good sequencing quality. Panels (E; before pre-processing) and (F; after pre-processing) indicate the percentage of adapter content along the read length; notably, panel (F) evidences that no significant adapter contamination (> 0.1%) was detected in any of the samples after the trimming step performed by the PreProcSEQ pipeline. These results confirm that the RNA-Seq data possess the necessary quality for robust immune repertoire analyses.


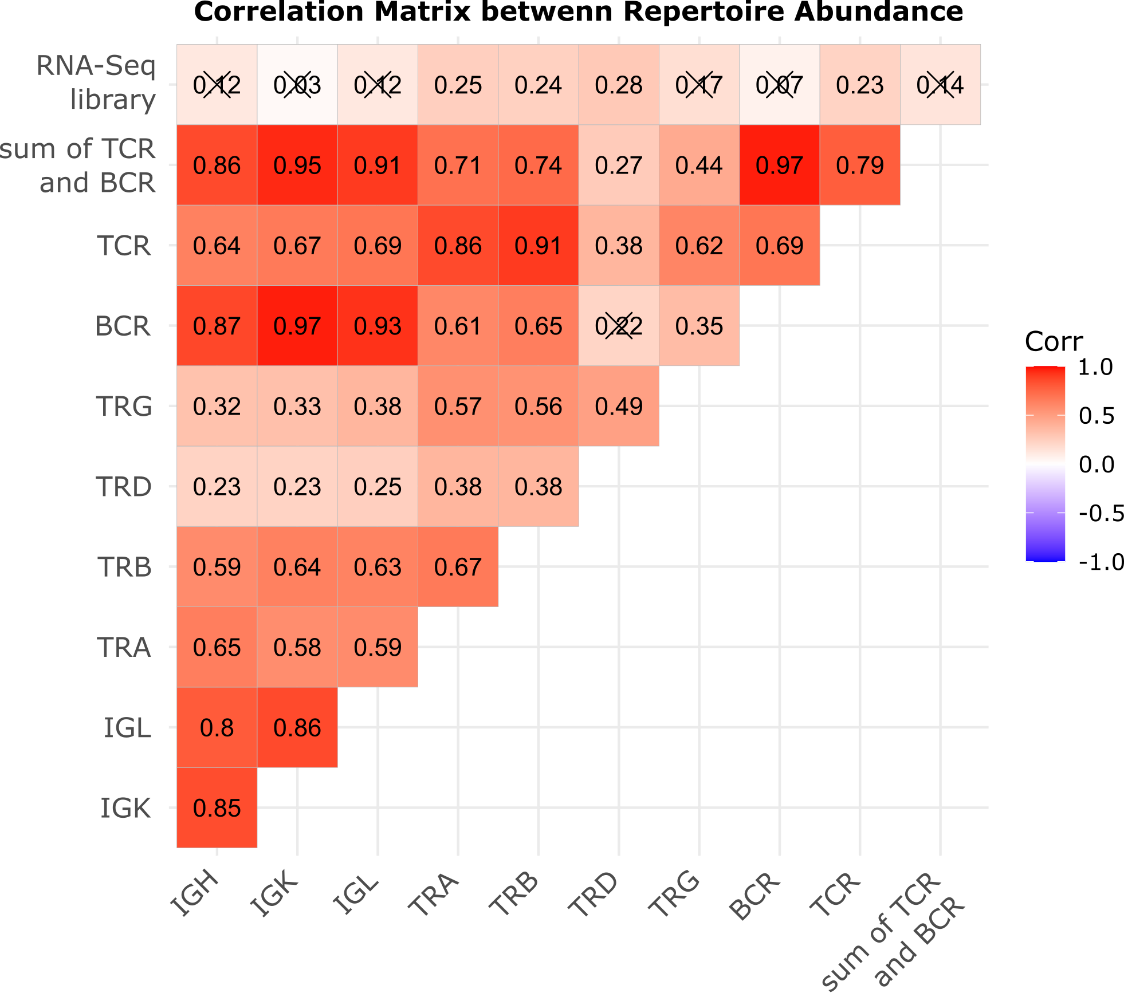


**FIGURE S2** Correlation Matrix Between Repertoire Abundance Metrics and RNA-Seq Library Size. This is a heatmap displaying the Spearman correlation coefficients between abundance counts for each individual receptor chain (IGH, IGK, IGL, TRA, TRB, TRD, TRG), total BCR and TCR reads, the overall repertoire sum (Sum of TCR and BCR), and the total RNA-Seq sequencing library size for each case. The intensity and color of the cells represent the Spearman's rho correlation coefficient, ranging from blue (strong negative correlation, -1) to red (strong positive correlation, +1), according to the indicated color scale. Cells marked with an 'X' indicate non-significant correlations (p > 0.05). This analysis demonstrates the interrelationships among the abundances of different chains and, crucially, evaluates the dependence of repertoire metrics on the total sequencing depth (library size). It confirms that the abundance of BCR and TCR chains is not significantly affected by library size, as indicated by the low correlation coefficients in the first row ('RNA-Seq library').


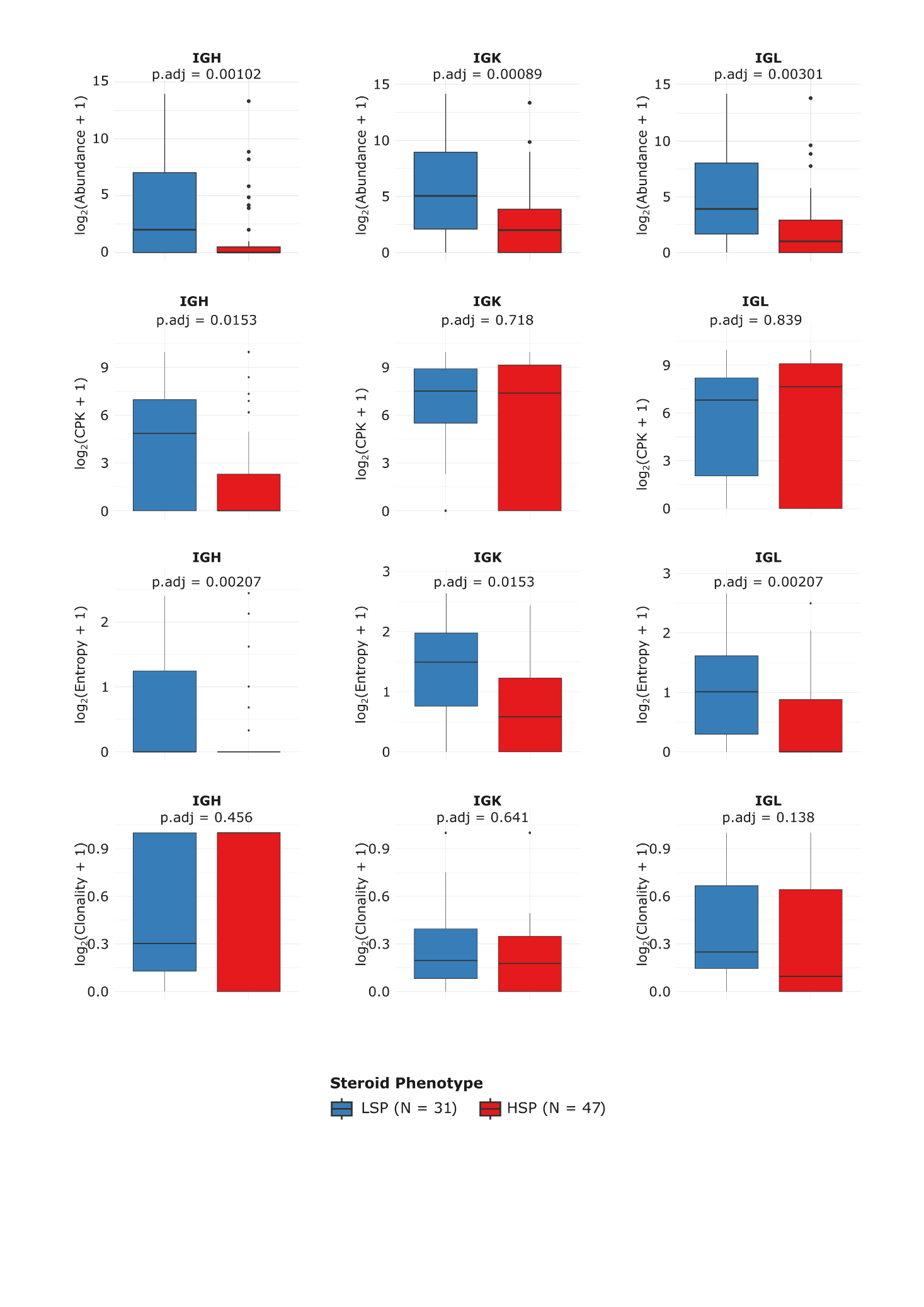
**FIGURE S3** Comparison of Immunological Repertoire Metrics for BCR Chains. Boxplots illustrate the comparison of Abundance, CPK (Clones per Kiloreads), Entropy (Shannon diversity), and Clonality metrics for B-cell receptor (BCR) chains (IGH, IGK, and IGL) between the Low Steroid Phenotype (LSP, N = 31, in blue) and High Steroid Phenotype (HSP, N = 47, in red) groups. For graphical representation, metric values were transformed to a log2(value+1) scale for normalization. The padj value (corrected for multiple comparisons via Benjamini-Hochberg FDR) is presented for each comparison. The Mann-Whitney test was used for group comparisons. The results demonstrate that the LSP group exhibits higher abundance and diversity for all BCR chains, and higher CPK for IGH, with statistically significant differences (p.adj < 0.05) for IGH, IGK, and IGL Abundance, IGH CPK, and IGH and IGL Entropy. Clonality, however, shows no significant differences.


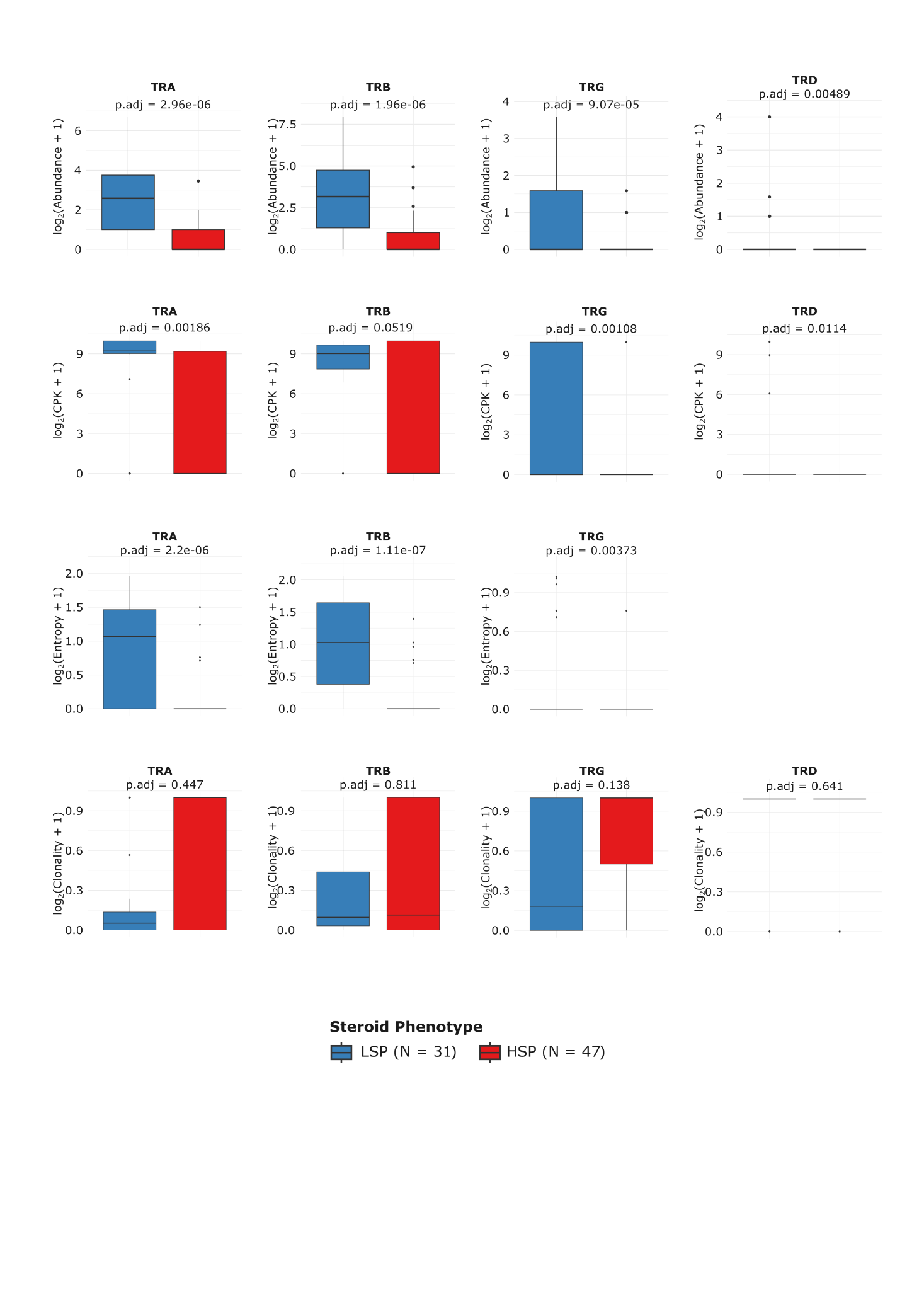


**FIGURE S4** Comparison of Immunological Repertoire Metrics for TCR Chains. Boxplots illustrate the comparison of Abundance, CPK (Clones per Kiloreads), Entropy (Shannon diversity), and Clonality metrics for T-cell receptor (TCR) chains (TRA, TRB, TRD, and TRG) between the Low Steroid Phenotype (LSP, N = 31, in blue) and High Steroid Phenotype (HSP, N = 47, in red) groups. For graphical representation, metric values were transformed to a log2(value+1) scale for normalization. The padj value (corrected for multiple comparisons via Benjamini-Hochberg FDR) is presented for each comparison. The Mann-Whitney test was used for group comparisons. Entropy for TRD could not be calculated due to the low detection frequency of this chain, especially in the HSP group. The results indicate that the LSP group shows significantly higher Abundance, CPK, and Entropy for most TCR chains (p.adj < 0.05), with the notable exception of the TRB chain which did not show a significant difference for CPK, and TRD which had limited data. Clonality does not exhibit significant differences between the groups.

**
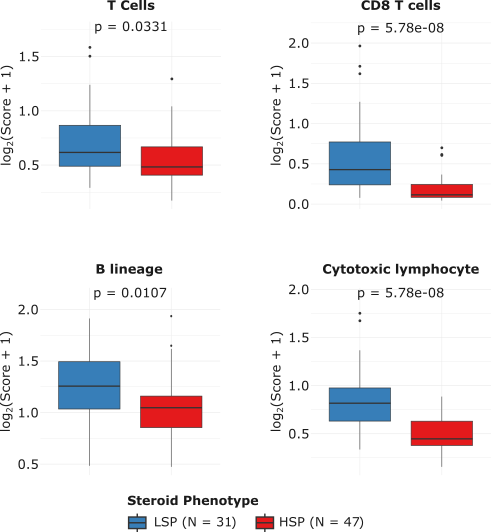
**

**FIGURE S5** Comparison of MCP-counter scores for T cells, CD8 cells, B cell lineage, and cytotoxic lymphocytes. Boxplots illustrate the comparison of T and B cell deconvolution scores between the low steroid phenotype (LSP, N = 31, in blue) and high steroid phenotype (HSP, N = 47, in red) groups. For graphical representation, metric values ​​were transformed to a log2(value + 1) scale for normalization. The p-value is presented for each comparison. The Mann-Whitney test was used for group comparisons.

**
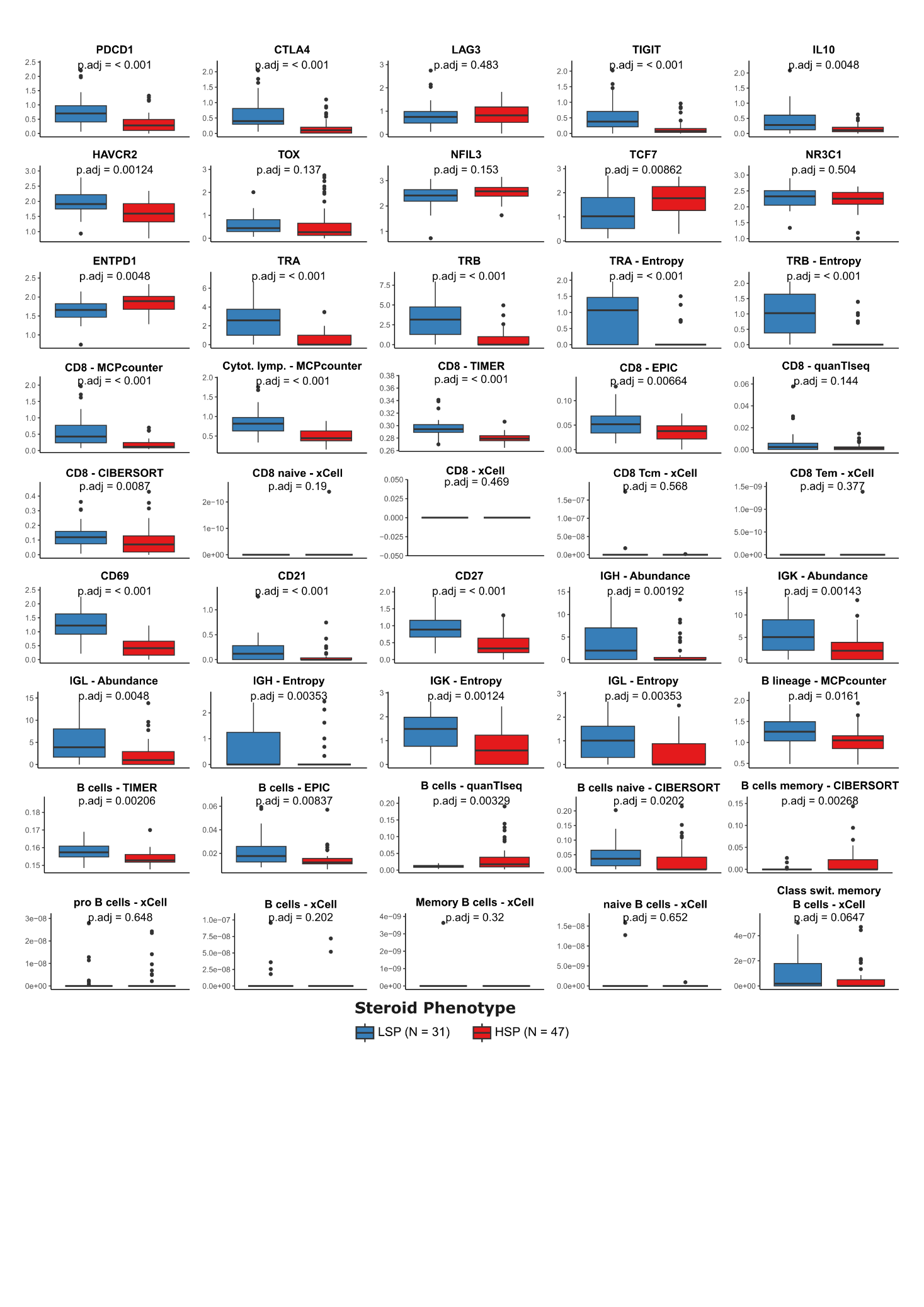
**

**FIGURE S6** Comparison of MCP-counter scores for T cells, CD8 cells, B cell lineage, and cytotoxic lymphocytes. Boxplots illustrate the comparison of T and B cell deconvolution scores between the low steroid phenotype (LSP, N = 31, in blue) and high steroid phenotype (HSP, N = 47, in red) groups. For graphical representation, metric values ​​were transformed to a log2(value + 1) scale for normalization. The p-value is presented for each comparison. The Mann-Whitney test was used for group comparisons.
